# Sex, Neural Networks, and Behavioral Symptoms Among Adolescents With Multisite Pain

**DOI:** 10.1001/jamanetworkopen.2025.5364

**Published:** 2025-04-16

**Authors:** Esmeralda Hidalgo-Lopez, Tristin Smith, Mike Angstadt, Hannah C. Becker, Andrew Schrepf, Daniel J. Clauw, Steven E. Harte, Mary M. Heitzeg, Jodi A. Mindell, Chelsea M. Kaplan, Adriene M. Beltz

**Affiliations:** 1 Department of Psychology, University of Michigan, Ann Arbor; 2 Chronic Pain and Fatigue Research Center, University of Michigan Medical School, Ann Arbor; 3 Department of Psychiatry, University of Michigan, Ann Arbor; 4 Department of Psychology, Saint Joseph’s University, Philadelphia, Pennsylvania; 5 Division of Pulmonary and Sleep Medicine, Children’s Hospital of Philadelphia, Philadelphia, Pennsylvania

## Abstract

**Importance:**

Multisite pain disproportionately affects females starting in adolescence and is associated with central nervous system dysregulation. Understanding the heterogeneity of underlying neural networks and behavioral symptoms is essential.

**Objective:**

To characterize sex-related resting-state neural networks and co-occurring symptoms, including sleep and behavioral problems, in youth with multisite pain.

**Design, Setting, and Participants:**

This cross-sectional analysis leverages the 2-year follow-up data from the Adolescent Brain and Cognitive Development Study. A total of 684 youth aged 11 to 12 years with multisite pain were compared with 1368 youth with no pain or with regional pain, matched by pubertal status, handedness, and race and ethnicity. Data were collected from July 2018 to February 2021 and released October 2021. Data were analyzed from June 2023 to July 2024.

**Exposure:**

Youth-reported number of painful regions during the last month classified into multisite (≥3), regional (1-2), and no pain groups.

**Main Outcomes and Measures:**

Sex-stratified group iterative multiple model estimation was used for sparse network estimation of regions from the salience network (SLN), sensorimotor network (SMN), and default mode network (DMN). Individual within-network and between-network densities were calculated. Symptoms were behavioral problems and sleep disturbances. Sex-stratified differences in network densities and symptoms were examined between groups. Associations between brain networks and co-occurring symptoms were explored.

**Results:**

Of 2052 participants (1044 [50.88%] female), mean (SD) pubertal status was 2.23 (0.65) and mean (SD) age was 12.02 (0.66) years; 25 (1.22%) were Asian, 149 (7.26%) were Black, 361 (17.59%) were Hispanic, 1307 (63.69%) were White, and 210 (10.23%) were other race or ethnicity. A total of 1646 participants (80.21%) were right-handed, 100 (4.87%) were left-handed, and 306 (14.91%) were ambidextrous. Multisite pain was associated with lower within-SMN connectivity in male (*F*_2,1005_ = 61.40; η^2^ = 0.11; false discovery rate [FDR] *P* < .001) and female (*F*_2,1041_ = 13.38; η^2^ = 0.03; FDR *P* < .001) participants and was associated with greater behavioral problems in male (*F*_2,918_ = 28.12; η^2^ = 0.04; FDR *P* < .001) and female (*F*_2,945_ = 9.12; η^2^ = 0.02; FDR *P* < .001) participants compared with the subgroup with no pain. Male participants with multisite pain had heightened DMN-SMN connectivity (*F*_2,1005_ = 3.55; η^2^ = 0.007; FDR *P* = .04). Female participants with multisite pain had heightened sleep disturbances (*F*_2,1039_ = 10.64; η^2^ = 0.02; FDR *P* = .002), partially explained by reduced within-SMN connectivity (indirect effect estimate, 0.15; 95% CI, 0.03-0.34).

**Conclusions and Relevance:**

In this cross-sectional study of 2052 adolescents, sex-related neurophysiological mechanisms were associated with multisite pain. Brain connectivity partially explained the sleep-pain association in female participants only. On replication and evidence of persistence, these findings suggest that female adolescents with pain may especially benefit from interventions targeting sleep disturbances.

## Key Points

**Question:**

Are there sex-associated alterations in resting-state neural connectivity and co-occurring symptoms in adolescents with multisite pain?

**Findings:**

In this cross-sectional study including 2052 adolescents, male and female participants with multisite pain had significantly reduced sensorimotor network connectivity and greater behavioral symptoms than those with no pain. Male adolescents with multisite pain exhibited heightened between-network connectivity, whereas female adolescents had increased sleep disturbances, partially explained by reduced sensorimotor network connectivity.

**Meaning:**

These findings suggest that understanding sex differences in neural networks and behavioral symptoms in adolescents with multisite pain is potentially helpful to developing more personalized and effective early treatment strategies.

## Teaser

This cross-sectional study characterizes resting-state functional connectivity in the salience, sensorimotor, and default mode networks of female and male adolescents with no pain, regional pain, and multisite pain.

## Introduction

Sex differences in pain are large and unmistakable: the burden of pain has been reported to disproportionately impact females.^1^ This divergence in pain experiences coincides with puberty,^2,3^ which marks the beginning of adolescence, and the largest sex difference is apparent in the prevalence of multisite pain (25.5% among female and 15.1% among male adolescents).^4^ Once established, the everyday consequences of adolescent pain are severe; pain is associated with poor school performance, activity limitations, and poor family functioning.^5,6^ Unsurprisingly, adolescent pain is often a harbinger of future problems, including increased risk of chronic pain and psychopathology in adulthood.^7,8,9^

Multisite pain is indicative of nociplastic pain, which is associated with altered pain processing within the central nervous system.^10,11^ Youth with multisite pain often experience co-occurring behavioral symptoms, including sleep disturbances, memory and attention deficits, and depressed mood. In fact, sleep disturbances and attentional issues seem to precede the development of multisite pain,^12^ potentially marking a neurobiological vulnerability to nociplastic pain. Supporting this, altered neural functional connectivity in the salience network (SLN), sensorimotor network (SMN), and default mode network (DMN) precedes the development of multisite pain in youth.^13^ Many studies in adults and emerging studies in youth indicate that individuals with multisite pain exhibit changes in these functional brain networks.^14,15,16^ For instance, reduced sensorimotor connectivity has been observed in adults and youth with fibromyalgia.^16,17^

Adolescent research on sex-related pain mechanisms that links behavior and the brain remains scarce. Puberty prompts dramatic changes in behavior and biology,^18,19^ with many sex-specific aspects, not only including neuroendocrine processes but also timing, with female youth maturing before male youth.^20^ Relevant to pain, female youth have more sleep difficulties and shorter sleep durations than male youth,^21,22^ and there are sex-related changes in neural reorganization, including regions and networks relevant to pain, during adolescence. For example, pubertal hormones contribute to the maturation of the inferior parietal cortex, a hub in the DMN implicated in pain processing.^23,24^

However, very few neuroimaging studies of pain examine sex differences. Although pain is more common in female than male youth, it can be life-altering for anyone who experiences it. Because most studies of patients with chronic pain use female-only or mixed-sex samples that statistically covary sex (thereby ignoring or misrepresenting sex-related effects^25^), it is unclear whether the extant literature–or treatments stemming from it–are relevant for male youth. To our knowledge, there are no studies examining the neurobiology of pain in male youth specifically. This problem is exacerbated in adolescent studies, which also have relatively small samples.^16,26^ Moreover, standard metrics of group-level functional connectivity, such as mean differences between patients and controls, may obscure potentially meaningful variation in individual brain networks (at best) or distort group-level inferences (at worst).^27,28^ Notably, pain and adolescence are both heterogeneous phenomena, and mathematical theorems show that mean findings cannot accurately reflect individual neurobiology.^29^ Hence, capturing the heterogeneous functional connectivity across youth is paramount to understanding the neural basis of pain and its links to behavioral symptoms; this will be key to future precision medicine efforts.

Our study leverages the Adolescent Brain Cognitive Development (ABCD)^30,31^ dataset and person-specific network analyses to accurately characterize resting-state functional connectivity in the SLN, SMN, and DMN of youth with no pain, regional pain, and multisite pain. Further, we examine subgroup differences in commonly co-occurring behavioral symptoms (eg, attention, sleep disturbances) and their association with brain connectivity. Owing to the qualitative nature of sex differences in pain, we conduct analyses separately for male and female youth.^32^ We hypothesize that brain networks would be heterogeneous and yet that female adolescents with multisite pain would show reduced connectivity within the SMN^16^ and heightened connectivity within and between other networks.^13,15^ We did not hypothesize about connectivity in male youth, owing to limited relevant literature. We also hypothesize that both male and female youth with multisite pain would have heightened sleep disturbances and behavioral problems (attentional, internalizing, and externalizing).^11,12^ Finally, we explore associations between behavioral symptoms and brain networks.

## Methods

### Participants

This cross-sectional study uses neuroimaging, and youth-reported and parent-reported questionnaires from the ABCD Study^30,31^ year-2 follow-up visit, when participants were aged 11 to 12 years. Data were collected from July 2018 to February 2021 (release 4.0 occurred October 2021).^33^ Participants provided written informed consent (parents) and assent (youth), conforming to the procedures of each of 21 sites’ institutional review boards. Detailed protocols of recruitment,^30^ assessment,^31^ and imaging^34^ have been published. This study adheres to the Strengthening the Reporting of Observational Studies in Epidemiology (STROBE) reporting guideline for cross-sectional studies.

**Figure 1.**
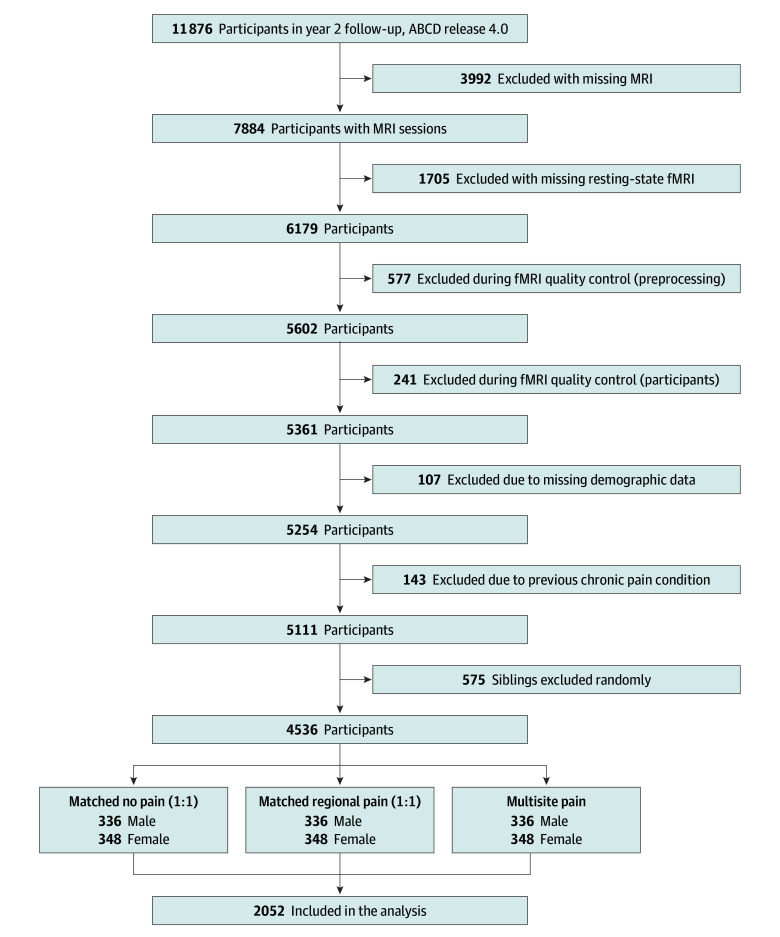
Flow Diagram Showing Exclusionary Criteria and Final Sample Participants’ data were preprocessed if they had at least 1 usable T1- or T2-weighted magnetic resonance imaging (MRI) scan and at least 2 functional runs of rest. Preprocessing quality checks included functional MRI (fMRI) quality control (preprocessing), including visualization of functional images after coregistration, registration to Montreal Neurological Institute template space, and exclusion of participants with less than 4 minutes of low motion data; and fMRI quality control (participants), involving exclusion of participants with more than 20% censored volumes or less than 2 consecutive resting state runs. Participants were also excluded for missing relevant demographics or preexisting chronic pain conditions. Only 1 child per family of the same sex was included to avoid dependencies and was performed separately for the multisite subgroup and the rest of the sample to retain as many participants with multisite pain as possible. For the final sample, we identified 684 youth with multisite pain. We matched participants with multisite pain separately for male and female on pubertal status, handedness, and race and ethnicity to an identical number of participants with no pain and an identical number of participants with regional pain. A final sample of 2052 participants was included in the analyses.

Figure 1 shows a participant flow diagram and final sample demographics. Subgroups (with no pain, regional pain, or multisite pain) were defined by the pain assessment described in the Measures subsection. After identifying 684 participants (348 female) with multisite pain, we used the MatchIt R package, version 4.5.5 (R Program for Statistical Computing)^35^ to match each of these participants by pubertal status, handedness, and race and ethnicity (consisting of Asian, Black, Hispanic, White, and other [including American Indian or Alaska Native, Native Hawaiian or Other Pacific Islander, non-Hispanic multiracial, or some other race]) to a participant from the no pain subgroup and then a participant from the regional pain subgroup (eMethods in Supplement 1), producing a sample of 2052 participants (1044 female). There were no differences among subgroups on the matching variables (eTable 2 in Supplement 1).

### Measures

#### Pain Assessment

Youth reported whether they had had any aches or pains over the last month. If “yes,” they selected specific pain locations on a body map.^36^ We categorized the locations based on the 2016 American College of Rheumatology body map, which is validated in youth.^37^ We then divided participants into 3 subgroups: no pain (0 regions), regional pain (1-2 regions), and multisite pain (≥3 regions) (eMethods in Supplement 1).

#### Brief Problem Monitor

Youth completed the Brief Problem Monitor,^38^ which measures attention, internalizing, and externalizing symptoms. We focused on the sum score and explored subscales (eMethods in Supplement 1); possible scores ranged from 0 to 38, with higher scores reflecting more problems.

#### Sleep Disturbances Scale for Children

Parents completed the Sleep Disturbances Scale for Children, which includes 6 subscales evaluating different aspects of sleep disorders (eMethods in Supplement 1).^39^ We focused on the total score and explored subscales; possible scores ranged from 26 to 130, with higher scores reflecting more problems.

### Functional Magnetic Resonance Imaging Preprocessing

Resting-state functional magnetic resonance imaging (fMRI) with high spatial (2.4-mm isotropic) and temporal (repetition time, 800 ms) resolution was acquired in 4 separate runs (5 minutes each, 20 minutes total^34^) and 2 consecutive runs for each participant were concatenated for analyses. Preprocessing was performed using the fMRIPrep tool, version 1.5.0^40^; details are in eMethods in Supplement 1. Preprocessed data were visually inspected by study staff, supervised by one of us (M.A.) at 2 stages to ensure only high-quality data were included in analyses: after coregistration of functional to structural data and after registration of functional data to Montreal Neurological Institute space. Volumes exceeding a framewise displacement threshold of 0.5 mm were censored. Only participants with fewer than 20% censored volumes and with 2 consecutive resting state runs were included (Figure 1). Mean (SD) framewise displacement was 0.18 (0.07) mm, and there were no significant differences among subgroups (eTables 8 and 9 in Supplement 1).

### fMRI Analysis

**Figure 2.**
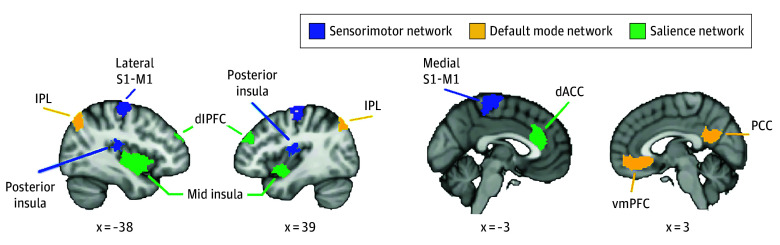
Preregistered Regions of Interest (ROIs) Defining Each Network Left and right hemisphere ROIs, grouped into sensorimotor network (SMN), default mode network (DMN), and salience network (SLN). Five ROIs constituted the SMN: bilateral lateral primary somatosensory and motor cortices (S1-M1), a medial S1-M1 region, and bilateral posterior insula. Four ROIs constituted the DMN: right ventromedial prefrontal cortex (vmPFC), right posterior cingulate cortex (PCC), and bilateral inferior parietal lobe (IPL). Five ROIs constituted the SLN: bilateral midinsula, left dorsal anterior cingulate cortex (dACC), and bilateral dorsolateral prefrontal cortices (dlPFC). Coordinates are shown in Montreal Neurological Institute space. Additional data are found in eMethods and eTable 1 in Supplement 1.

fMRI time series from 14 preregistered regions of interest (ROIs)^41^ in the 300-parcelled Schaefer atlas^42^ (Figure 2) were extracted from the preprocessed data to be entered into connectivity analyses with group iterative multiple model estimation (GIMME) (eMethods in Supplement 1). ROI selection was based on network characterizations in youth,^43,44^ regions exhibiting links to pain in youth,^13,16,45^ the proximity of selected regions (eg, limiting overlap), and regions exhibiting links to chronic pain in adults (if limited information from youth)^46,47^ (eTable 1 in Supplement 1). As recommended,^28^ the multiband time series were downsampled by half for GIMME, resulting in time series lengths of 375 time points for each participant.

### Person-Specific Functional Connectivity Mapping With Confirmatory Subgrouping-GIMME

Person-specific functional connectivity mapping with confirmatory subgrouping GIMME (CS-GIMME) (gimme R package, version 0.7.15)^48,49^ was performed, using a priori subgroups (no pain, regional pain, or multisite pain), separately for male and female participants. A comprehensive depiction of the details of CS-GIMME appears in eFigure 1 in Supplement 1. GIMME is the optimal approach for estimating functional connectivity in heterogenous data,^27^ with validations through large-scale simulations and utility evidenced in clinical applications.^28,50^ Generally, GIMME fits person-specific unified structural equation models by iteratively adding directed connections at the group, subgroup, and then individual levels.^51^ Connections are contemporaneous (same time point) or lagged (1 time point prior), and directionality matters. In our application, autoregressive relations (each ROI estimating itself at the next time point) were estimated^52^ as group-level priors, and then the group-level search iteratively estimated the sample-level connections using Lagrange multiplier tests to determine which connections, if added, would improve network fit for at least 75% of male or female participants. Then using group-level connections as priors, CS-GIMME iteratively estimated subgroup-level connections if they improved model fit for at least 51% of the subgroup (no pain, regional pain, or multisite pain), according to Lagrange multiplier tests. Finally using group-level and subgroup-level connections as priors, GIMME iteratively estimated individual-level connections until each network fit each participant’s data well, according to standard indices (2 of 4): root-mean-square error of approximation less than or equal to 0.05; standard root mean residual less than or equal to 0.05; comparative fit index greater than or equal to 0.95; and nonnormed fit index greater than or equal to 0.95.^27^ Finally, we characterized each person-specific network by (1) complexity, or number of connections in the entire network; (2) within-network densities, or number of connections between any 2 nodes of the same network (eg, SMN), relative to complexity; and (3) between-network densities, or number of connections between any 2 nodes of different networks (eg, SMN-SLN), relative to complexity.

### Statistical Analysis

Data were analyzed from June 2023 to July 2024. Analyses were performed separately for male and female participants in R, version 4.2.0.^53^ One-way analyses of variance, with post hoc Tukey honestly significant difference tests (R stats package, version 3.6.2) were used to assess differences in network densities (within- and between-network) and symptoms (behavior problems and sleep disturbances), among subgroups (no pain, regional pain, or multisite pain). Given our sex-specific matching on critical variables, no covariates were included, except for motion in sensitivity analyses. Following ABCD consortium guidelines,^54^ we also performed sensitivity analyses including scanner manufacturer (GE HealthCare Technologies Inc, Koninklijke Philips NV, and Siemens AG) as random effects (lme4 package in R, version 1.1-36). All *P* values were corrected for false discovery rate (FDR) for multiple comparisons across the 3 networks; 2-sided FDR *P* < .05 indicated statistical significance. We then examined associations between network densities and symptoms using bivariate correlations. Finally, only for variable pairs that showed subgroup differences, we tested whether the association between subgroup and symptoms occurred via network densities. A mediation analysis used the mediation1 function of R package MeMoBootR^55^ with 10 000 bootstrapped samples and 95% bias-corrected CIs to interpret indirect effect estimates, where an estimate was considered significant if the 95% CI did not include zero.^56^

## Results

The final sample included 2052 participants (1044 female [50.88%] and 1008 male [49.12%]) with mean (SD) pubertal status of 2.23 (0.65) and mean (SD) age of 12.02 (0.66) years. In terms of race and ethnicity, 25 participants (1.22%) were Asian; 149 (7.26%), Black; 361 (17.59%), Hispanic; 1307 (63.69%), White; and 210 (10.23%), other race or ethnicity. A total of 1646 participants (80.21%) were right-handed; 100 (4.87%), left-handed; and 306 (14.91%), ambidextrous. Descriptive statistics for demographic characteristics, network densities, and behavioral symptoms by sex and subgroup are in eTables 2 and 3 in Supplement 1.

### Person-Specific Network Maps

**Figure 3.**
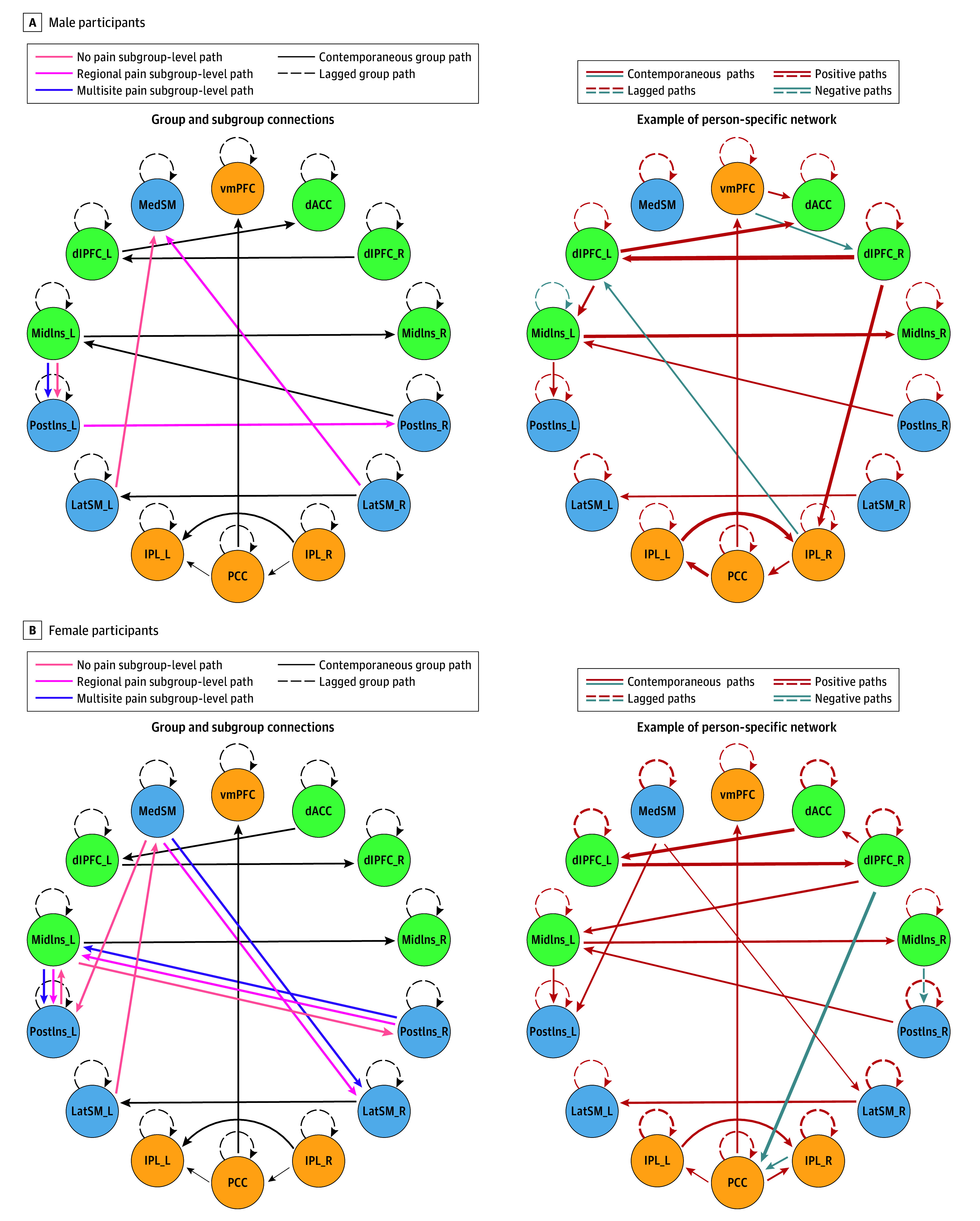
Connectivity Maps With Confirmatory Subgrouping Group Iterative Multiple Model Estimation Summary maps (left) show group- and subgroup-level connections. Of 23 group-level for males and 22 for females, 14 were autoregressions (estimated a priori, dashed circular lines), and 9 or 8 were contemporaneous. In person-specific maps for those with multisite pain (right) connections are directional with line thickness corresponding to magnitude. Of 23 group-level for males and 22 for females, 14 were autoregressions, and 9 for males and 8 for females were contemporaneous. dACC indicates dorsal anterior cingulate cortex; dlPFC, dorsolateral prefrontal cortex; IPL, inferior parietal lobe; LatSM, lateral somatosensory and motor cortex; MedSM, medial somatosensory motor cortex; MidIns, mid-insula; PostIs, posterior insula; PCC, posterior cingulate cortex; and vmPFC, ventromedial prefrontal cortex.

Each participant’s GIMME network converged normally and fit the data well (eTable 4 in Supplement 1). Figure 3 presents summary networks across male and female participants as well as illustrative person-specific networks for individuals of each sex with multisite pain (right panels). An extended figure with person-specific examples is in eFigure 2 in Supplement 1.

### Subgroup Differences in Network Densities

**Figure 4.**
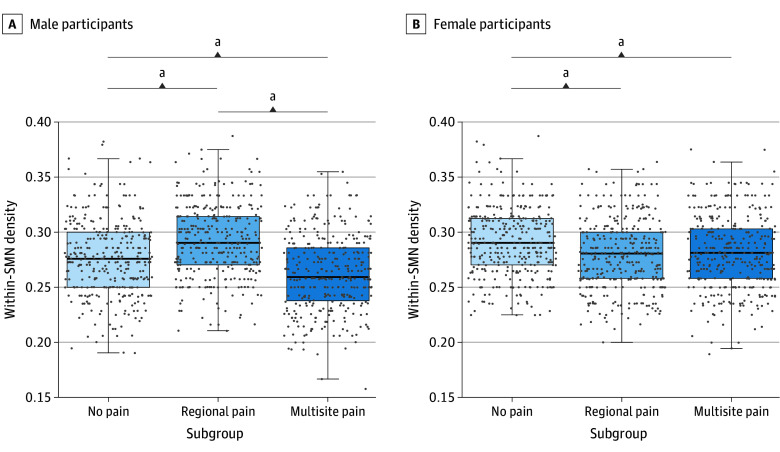
Differences in Sensorimotor Within-Network Connectivity Densities Among Subgroups With No Pain, Regional Pain, and Multisite Pain Stratified by Sex Differences in male and female participants in within–sensorimotor network (SMN) densities. Boxes indicate the IQR; data points, individual density; horizontal lines, median; and whiskers, 1.5 times the IQR. ^a^*P* < .001, pair-wise Tukey honestly significant difference comparisons.

Male participants with no pain and regional pain had subgroup-level contemporaneous connections within the SMN (Figure 3A). These connections were absent in the multisite pain subgroup, suggesting heightened heterogeneity. Accordingly, the multisite pain subgroup had lower within-SMN density compared with the regional or no pain subgroups (*F*_2, 1005_ = 61.40; η^2^ = 0.11; FDR *P* < .001) (Figure 4A). The multisite pain subgroup also had higher DMN density than the regional pain subgroup (*F*_2, 1005_ = 5.88; η^2^ = 0.01; FDR *P* = .005) and between the DMN-SMN than the no pain subgroup (*F*_2, 1005_ = 3.55; η^2^ = 0.007; FDR *P* = .04). Finally, the regional pain subgroup had lower SMN-SLN density relative to the no pain and multisite pain subgroups (*F*_2, 1005_ = 22.31; η^2^ = 0.04; FDR *P* < .001). Results are shown in eFigure 3 and eTable 5 in Supplement 1.

Female participants in the no pain subgroup had subgroup-level contemporaneous within-SMN connections (Figure 3B) that were absent in the other subgroups. In turn, the regional and multisite pain subgroups had a contemporaneous connection from the medial primary somatosensory and primary motor cortex (S1-M1) to the right lateral S1-M1 cortex. Accordingly, female participants in subgroups with pain (regional and multisite) had lower within-SMN density compared with those with no pain (*F*_2, 1041_ = 13.38; η^2^ = 0.03; FDR *P* < .001) (Figure 4B). Additionally, the multisite pain subgroup had higher DMN density than the no pain subgroup (*F*_2, 1041_ = 4.22; η^2^ = 0.008; FDR *P* = .02) (eFigure 3B in Supplement 1). No differences were found in between-network densities (eTable 5 in Supplement 1).

Sensitivity analyses examined whether results were robust to scanner manufacturer (random effects in linear mixed models) and in-scanner motion (covariates in analyses of covariance). Findings were consistent with the pattern of results shown in eFigure 3 and eTable 5 in Supplement 1 (see eTables 7 and 10 in Supplement 1).

### Subgroup Differences in Behavioral Symptoms and Associations With Network Densities

**Figure 5.**
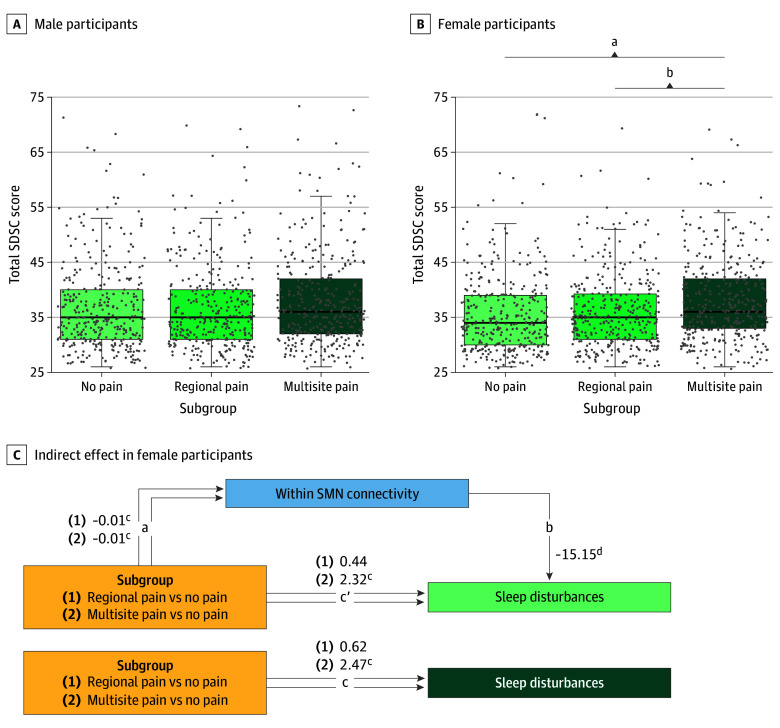
Differences in Sleep Disturbances Among Subgroups With No Pain, Regional Pain, and Multisite Pain Differences in Sleep Disturbances Scale for Children (SDSC) scores are stratified by sex (A and B). Scores range from 26 to 73, with higher scores indicating more problems. Boxes indicate the IQR; data points, individual density; horizontal lines, median; and whiskers, 1.5 times the IQR. Mediation analysis shows the indirect effect of sensorimotor network (SMN) density (blue) on the association between subgroup (yellow) and sleep disturbances (green) in female participants only (C). Unstandardized estimates of each of the paths are presented for regional pain vs no pain subgroups (1) and multisite pain vs no pain subgroups (2). Path a represents differences in within-SMN connectivity by subgroup; path b, association between sleep disturbances and within-SMN connectivity; path c (total), differences in sleep disturbances by subgroup; and path c’ (direct), differences in sleep disturbances by subgroup accounting for within-SMN density. Indirect effects are reported in text with 95% CIs. ^a^*P* < .001, pair-wise Tukey honestly significant difference comparisons. ^b^*P* = .003, pair-wise Tukey honestly significant difference comparisons. ^c^*P* < .001. ^d^*P* < .05.

Both male and female participants in the multisite pain subgroups had heightened total Brief Problem Monitor scores (eFigure 4 in Supplement 1) and all subscale scores (eTable 5 in Supplement 1). Only female participants in the multisite pain subgroup had higher scores for total sleep disturbances (F_2, 1039_ = 10.64; η^2^ = 0.02; FDR *P* = .002) (Figure 5B) and the Disorders of Initiating and Maintaining Sleep, Sleep-Wake Transition Disorders, and Disorders of Excessive Somnolence sleep disturbance subscales (eTable 5 in Supplement 1) than the other subgroups.

Only female participants showed a correlation of within-SMN density with behavioral symptoms, specifically an inverse correlation with total sleep disturbances (*r* = −0.08; 95% CI, −0.14 to −0.02; *P* = .01) (eTable 11 in Supplement 1). We further found evidence of an indirect effect with mediation analysis (Figure 5C). The unstandardized bias-corrected bootstrapped indirect effect for regional vs no pain was 0.17 (95% CI, 0.03-0.38), and for multisite vs no pain was 0.15 (95% CI, 0.03-0.34), suggesting that a statistically significant portion of the correlation between subgroup and sleep disturbances occurs through within-SMN density.

## Discussion

In what is to our knowledge the largest pediatric pain neuroimaging investigation to date, our cross-sectional study found shared and distinct underlying brain networks and co-occurring behavioral symptoms for male and female youth with multisite pain. Both male and female participants with multisite pain had reduced within-SMN connectivity; those with no pain had heightened within-SMN connectivity, including a connection between the left medial and lateral S1-M1 that may be protective. Male and female participants with multisite pain also had greater behavior problems than youth with no pain as well as indications of heightened DMN connectivity. Male participants with multisite pain uniquely showed heightened between-network connectivity (DMN-SMN), whereas female participants with multisite pain uniquely reported elevated sleep disturbances that were not only associated with SMN density, but were partially accounted for by it.

We uncovered these novel findings by combining person-specific neural network mapping (preventing averaging across unique neurobiological features)^29^ with sex stratification (reflecting sex as a biological variable best practices) in multimodal data.^32^ To date, most neuroimaging studies of pain in youth do not have enough power to adequately study the sexes separately^25^ or have focused exclusively on female youth.^16^ They also have not linked neural mechanisms to behavioral symptoms demonstrated to precede pain.^12^ Together, our findings begin to fill the critical knowledge gap concerning the ways in which sex differences in the adolescent brain^23,24^ and adult pain neurobiology^14,15^ matter for the neurodevelopment and everyday challenges associated with multisite pain in youth.

We intentionally studied male youth and found that those with multisite pain had reduced within-SMN connectivity (vs those with regional or no pain) and heightened DMN connectivity (vs those with regional pain). These patterns were largely similar to those of female participants with multisite pain and mirror the (predominantly female) literature in adulthood.^14,16,17,57^ In contrast, male participants showed sex-specific differences in between-network connectivity: those with multisite pain had greater DMN-SMN connectivity than male participants with no pain. Alongside reduced within-SMN connectivity, heightened DMN-SMN connectivity is observed in many nociplastic pain conditions.^11,58,59^ There were also some unique neural features of male participants with regional pain (perhaps reflecting the transition to multisite pain), so longitudinal work is needed.

In female youth with multisite pain relative to those with regional or no pain, we expectedly observed reduced within-SMN (consistent with research in female youth with juvenile fibromyalgia^16^) and greater DMN connectivity.^14,57^ Respectively, this connectivity pattern at rest might reflect weaker sensorimotor integration,^14,16^ with increased higher-order interoceptive awareness.^57^ Female participants with multisite pain also experienced greater sleep disturbances.^60^ Poor sleep can result in multiple forms of diminished well-being^61^ and is an important risk factor for the development and persistence of pain.^12,62^ Pediatric research on sex differences in the association between sleep and pain is sparse and mixed, with some studies reporting links between poor sleep quality and pain onset only in male youth,^63^ and others finding links with persistent headaches^64^ and musculoskeletal pain only in female youth.^65,66^ Discrepant findings are likely due to methodological variations across studies that our large matched sex-stratified approach mitigate.

Importantly, and uniquely for female adolescents, the association between pain and sleep disturbances was partially explained by reduced within-SMN connectivity, elucidating the importance of brain connectivity changes underlying the pain-sleep association, especially for female youth. This refines previous work in a combined pediatric and adult sample (predominantly female) in which chronic pain also presented with diminished sensory circuit connectivity in brain areas that show an effect of sleep deprivation.^67,68^

We found that behavior problems (internalizing, externalizing, and attention) were elevated in multisite pain for both sexes, consistent with a previous study.^69^ Although some work has shown that male youth with multisite or chronic pain display heightened externalizing problems and female youth display heightened internalizing problems,^70,71^ these patterns parallel sex differences detected in youth without pain,^72,73^ challenging the unique role of pain. We also found heightened attention problems in both male and female participants with multisite pain. A recent study showed that attentional issues in youth are robustly associated with new multisite pain 1 year later,^12^ and it is well-established that problems with cognition are reported across nociplastic pain conditions.^11,74^ Somewhat surprisingly, we did not find significant differences between no, regional, and multisite pain subgroups in between-network density in female participants. This may be due to differences in connectivity methods used in this and past studies. We also did not find associations between behavioral problems and network densities in either sex, which is not terribly surprising, given the elusive neural networks underlying such problems,^75^ and our focus on networks underpinning pain. Future studies using functional connectivity approaches well-suited to detect relations among many multifaceted networks should specifically examine the relationship between pain and behavioral problems in youth.

### Strengths and Limitations

We used cross-sectional data from the ABCD Study, using the newly added youth-reported measure of pain on a well-validated body map in the largest sample of its kind; this is an important study strength.^13^ However, the measure asks about pain experienced over the past month, so whether the pain reported was acute or chronic, or whether participants sought treatment remains unknown. Although multisite pain is suggestive of nociplastic pain mechanisms,^11^ longitudinal studies are required to assess whether it becomes chronic. Moreover, the cross-sectional data do not permit inferences about directionality in the indirect effect analysis. The matching procedure, however, ensured that the subgroups were compared only in terms of differences in number of pain sites. The novel GIMME approach also allowed us to accurately analyze heterogeneous individualized neural networks, but a drawback is the relatively limited number of brain regions that can be included.^28^ Accordingly, the brain regions investigated were established a priori and were preregistered (eMethods in Supplement 1).^41^ Finally, whereas the pain and behavioral problems were reported by youth, parents or guardians reported their child’s sleep disturbances. As parents often underreport disturbances,^76^ our findings, despite their significance, may be underestimates.

## Conclusions

In this cross-sectional study, findings illuminate the distinct brain and behavioral underpinnings of multisite pain in male and female adolescents, emphasizing that sleep may be an important risk marker for female youth. Understanding the role of sex in the intricate associations among brain function, behavior, and pain in the critical developmental period when behavior problems peak and when sleep disturbances and chronic pain emerge is imperative for uncovering the neurobiological origins of chronic pain and for ultimately informing individualized therapies.

## Notes

Supplement 1. **eMethods.** Matching Procedure, Measures, fMRIPrep Preprocessing, Time Series Extraction, and ROI Selection **eTable 1.** Definitions of Regions of Interest Used in Functional Connectivity Analyses According to Their Schaefer Atlas Nomenclature **eTable 2.** Matched Sample Demographics, Showing Percentages by Sex and Subgroups **eFigure 1.** Schematic Summary of the Analytical Design, Including a Flow Chart Describing GIMME, Its Model Fitting Procedures, and Graphical Depiction of Present Results **eTable 3.** Descriptive Statistics for Network Densities and Symptom Domains by Sex and Subgroups (No Pain, Regional Pain, and Multisite Pain) **eTable 4.** GIMME Model Fit Indices Averaged by Sex and Subgroups **eFigure 2.** CS-GIMME Models for Each Subgroup by Sex, With Examples of Person-Specific Maps **eFigure 3.** Differences in Network Connectivity Densities Between No Pain, Regional Pain, and Multisite Pain Subgroups Stratified by Sex **eTable 5.** One-Way Analysis of Variance Results for Network Densities and Symptom Domains (Behavior Problems and Sleep Disturbances) **eTable 6.** Number of Participants Scanned by Each MRI Manufacturer by Sex and Subgroups (No Pain, Regional Pain, and Multisite Pain) **eTable 7.** Linear Mixed Models Results for Subgroup Differences in Network Densities With Scanner Manufacturer (3 Groups) as Random Effects **eTable 8.** Descriptive Statistics for Movement Parameters by Sex and Subgroups (No Pain, Regional Pain, and Multisite Pain) **eTable 9.** One-Way Analysis of Variance Results for Differences Between Subgroups in Movement Parameters **eFigure 4.** Differences in Behavioral Problems Among No Pain, Regional Pain, and Multisite Pain Subgroups by Sex **eTable 10.** Analysis of Covariance Results for Subgroup Differences in Network Densities With Movement Parameters as Covariates **eTable 11.** Correlations Between Network Densities and Symptom Domains
eReferences

Supplement 2.
Data Sharing Statement
