# Supplementary material for: Sex, Neural Networks, and Behavioral Symptoms Among Adolescents With Multisite Pain
Source: JAMA Netw Open. 2025 Apr 16;8(4):e255364. doi: 10.1001/jamanetworkopen.2025.5364 (PMC12004202; doi:10.1001/jamanetworkopen.2025.5364)
Supplement: Supplement 1. — eMethods. Matching Procedure, Measures, fMRIPrep Preprocessing, Time Series Extraction, and ROI Selection eTable 1. Definitions of Regions of Interest Used in Functional Connectivity Analyses According to Their Schaefer Atlas Nomenclature eTable 2. Matched Sample Demographics, Showing Percentages by Sex and Subgroups eFigure 1. Schematic Summary of the Analytical Design, Including a Flow Chart Describing GIMME, Its Model Fitting Procedures, and Graphical Depiction of Present Results eTable 3. Descriptive Statistics for Network Densities and Symptom Domains by Sex and Subgroups (No Pain, Regional Pain, and Multisite Pain) eTable 4. GIMME Model Fit Indices Averaged by Sex and Subgroups eFigure 2. CS-GIMME Models for Each Subgroup by Sex, With Examples of Person-Specific Maps eFigure 3. Differences in Network Connectivity Densities Between No Pain, Regional Pain, and Multisite Pain Subgroups Stratified by Sex eTable 5. One-Way Analysis of Variance Results for Network Densities and Symptom Domains (Behavior Problems and Sleep Disturbances) eTable 6. Number of Participants Scanned by Each MRI Manufacturer by Sex and Subgroups (No Pain, Regional Pain, and Multisite Pain) eTable 7. Linear Mixed Models Results for Subgroup Differences in Network Densities With Scanner Manufacturer (3 Groups) as Random Effects eTable 8. Descriptive Statistics for Movement Parameters by Sex and Subgroups (No Pain, Regional Pain, and Multisite Pain) eTable 9. One-Way Analysis of Variance Results for Differences Between Subgroups in Movement Parameters eFigure 4. Differences in Behavioral Problems Among No Pain, Regional Pain, and Multisite Pain Subgroups by Sex eTable 10. Analysis of Covariance Results for Subgroup Differences in Network Densities With Movement Parameters as Covariates eTable 11. Correlations Between Network Densities and Symptom Domains eReferences [file jamanetwopen-e255364-s001.pdf]

Supplementary Online Content

Hidalgo-Lopez E, Smith T, Angstadt M, et al. Sex, neural networks, and behavioral symptoms among adolescents with multisite pain. *JAMA Netw Open.* 2025;8(4):e255364. doi:10.1001/jamanetworkopen.2025.5364

eMethods. Matching procedure, Measures, fMRIPrep Preprocessing, Time Series Extraction, and ROI Selection

eTable 1. Definitions of Regions of Interest Used in Functional Connectivity Analyses According to Their Schaefer Atlas Nomenclature

eTable 2. Matched Sample Demographics, Showing Percentages by Sex and Subgroups

eFigure 1. Schematic Summary of the Analytical Design, Including a Flow Chart Describing GIMME, Its Model Fitting Procedures, and Graphical Depiction of Present Results

eTable 3. Descriptive Statistics for Network Densities and Symptom Domains by Sex and Subgroups (No Pain, Regional Pain, and Multisite Pain)

eTable 4. GIMME Model Fit Indices Averaged by Sex and Subgroups

eFigure 2. CS-GIMME Models for Each Subgroup by Sex, With Examples of Person-Specific Maps

eFigure 3. Differences in Network Connectivity Densities Between No Pain, Regional Pain, and Multisite Pain Subgroups Stratified by Sex

eTable 5. One-Way Analysis of Variance Results for Network Densities and Symptom Domains (Behavior Problems and Sleep Disturbances)

eTable 6. Number of Participants Scanned by Each MRI Manufacturer by Sex and Subgroups (No Pain, Regional Pain, and Multisite Pain)

eTable 7. Linear Mixed Models Results for Subgroup Differences in Network Densities With Scanner Manufacturer (3 Groups) as Random Effects

eTable 8. Descriptive Statistics for Movement Parameters by Sex and Subgroups (No Pain, Regional Pain, and Multisite Pain)

eTable 9. One-Way Analysis of Variance Results for Differences Between Subgroups in Movement Parameters

eFigure 4. Differences in Behavioral Problems Among No Pain, Regional Pain, and Multisite Pain Subgroups by Sex

eTable 10. Analysis of Covariance Results for Subgroup Differences in Network Densities With Movement Parameters as Covariates

eTable 11. Correlations Between Network Densities and Symptom Domains

eReferences.

This supplementary material has been provided by the authors to give readers additional information about their work.

© 2025 Hidalgo-Lopez E et al. *JAMA Network Open*.

Methods. Matching procedure, Measures, fMRIPrep Preprocessing, Time Series Extraction, and ROI Selection

Matching procedure

Within each sex, optimal pair matching was used for *pubertal status*, *handedness,* and *race/ethnicity* to optimize the sum of the absolute pair distances in the matched sample (see next section for details on these measures). The propensity scores used to compute the distance between units were estimated by the default "glm" option.[1,2]

Measures

Pain Assessment: Youth were asked whether they had had any aches or pains over the last month. If they answered "yes", they were shown a body map depicting 75 regions and asked to select the specific locations where they experienced pain. This body map represents a modified version of the Collaborative Health Outcomes Information Registry (CHOIR), plus one additional region, the mouth.[3] To stay consistent with the CHOIR body map, we excluded the mouth from our analyses. Next, we mapped these 74 regions onto the 2016 American College of Rheumatology body map as this has been validated in children[4] and to more accurately characterize multisite pain. We divided participants into 3 subgroups: *no pain* (0 endorsed regions), *regional* pain (1-2 endorsed regions), and *multisite* pain (3 or more endorsed regions).

Brief Problem Monitor: Children completed the Brief Problem Monitor (BPM),[5] which contains 19 items divided into 3 subscales: a) attention, b) internalizing, and c) externalizing symptoms, with higher scores reflecting more behavioral problems. The BPM is a psychometrically validated scale in which each item has a rating scale ranging from 0 (*Not true*) to 2 (*Very true*),[6] and requires all items to be answered in order to calculate a sum score. *BPM* was operationalized as the sum score in a continuous variable and used for all participants except for 87 males and 96 females who had missing data.

Sleep Disturbances Scale for Children: Parents completed the Sleep Disturbance Scale for Children (SDSC), which contains 26 items across 6 subscales: a) disorders of initiating and maintaining sleep, b) sleep breathing disorders, c) disorders of arousal, d) sleep-wake transition disorders, e) disorders of excessive somnolence, and f) sleep hyperhidrosis.[7] The overall total score is the sum of the 6 subscales scores. Questions are answered as either *Never*, *Occasionally* (once or twice per month or less), *Sometimes* (once or twice per week), *Often* (3-5 times per week), and *Always* (daily). These items were answered pertaining to the past 6 months of a child's life. Overall total scores range from 26 to 130, with higher scores denoting more sleep disturbances. Data were included for all participants except for 1 male and 2 females that had missing data.

Pubertal status: The parent-reported Pubertal Development Scale items with puberty status range of (1.0-4.0) were used.[8] Consistent with prior research, we utilized the average score of parent-rated items that included five physical characteristics. Parents of both boys and girls answered questions regarding growth spurts, body hair, and skin changes. Parents of boys answered a question on facial hair and voice deepening. Parents of girls answered questions on breast development and menarche. Items are answered from 1-4 where 1 represents *'has not yet begun'*, 2 represents *'has barely started'*, 3 represents "*is definitely underway*", and 4 represents *"seems complete".* Higher scores are indicative of later stage puberty. A score was calculated only when four or more (out of five) items were responded and used as a continuous variable.

Handedness: Child handedness was coded as either left-handed, right-handed or mixed based on a composite of participants rating, using a short form of the Edinburgh Handedness Inventory.[9] Participants were asked to report if they always or usually used their right/left hand, or if they used both hands equally when a) writing, b) throwing, c) using their toothbrush, and d) using a spoon. The response choices included *Always Right*, *Usually Right*, *Both Equally*, *Usually Left*, and *Always Left*. Those answer choices correspond respectively to the numerical values 100, 50, 0, -50, and -100. We used the average composite score as a categorical variable, with 3 categories: right-handed if the mean of these items were higher than 60; left-handed when the mean was lower than -60; and mixed-handed for any score in between.

Race/ethnicity: Parents reported child race/ethnicity at baseline, choosing among White, Black, Hispanic, Asian, and other.

© 2025 Hidalgo-Lopez E et al. *JAMA Network Open*.

fMRIPrep Preprocessing

The entire data pipeline was run through automated scripts on the University of Michigan's high-performance cluster. The following was generated automatically by fMRIPrep software and is copied here unchanged:

Results included in this manuscript come from preprocessing performed using *fMRIPrep* 1.5.0 (Esteban, Markiewicz, et al. (2018); Esteban, Blair, et al. (2018); RRID:SCR_016216), which is based on *Nipype* 1.2.2 (Gorgolewski et al. (2011); Gorgolewski et al. (2018); RRID:SCR_002502).

Anatomical data preprocessing

The T1-weighted (T1w) image was corrected for intensity non-uniformity (INU) with N4BiasFieldCorrection (Tustison et al. 2010), distributed with ANTs 2.2.0 (Avants et al. 2008, RRID:SCR_004757), and used as T1w-reference throughout the workflow. The T1w-reference was then skull-stripped with a *Nipype* implementation of the antsBrainExtraction.sh workflow (from ANTs), using OASIS30ANTs as target template. Brain tissue segmentation of cerebrospinal fluid (CSF), white-matter (WM) and gray-matter (GM) was performed on the brain-extracted T1w using fast (FSL 5.0.9, RRID:SCR_002823, Zhang, Brady, and Smith 2001). Brain surfaces were reconstructed using recon-all (FreeSurfer 6.0.1, RRID:SCR_001847, Dale, Fischl, and Sereno 1999), and the brain mask estimated previously was refined with a custom variation of the method to reconcile ANTs-derived and FreeSurfer-derived segmentations of the cortical gray-matter of Mindboggle (RRID:SCR_002438, Klein et al. 2017). Volume-based spatial normalization to one standard space (MNI152NLin6Asym) was performed through nonlinear registration with antsRegistration (ANTs 2.2.0), using brain-extracted versions of both T1w reference and the T1w template. The following template was selected for spatial normalization: *FSL's MNI ICBM 152 non-linear 6th Generation Asymmetric Average Brain Stereotaxic Registration Model* [Evans et al. (2012), RRID:SCR_002823; TemplateFlow ID: MNI152NLin6Asym].

Functional data preprocessing

For each of the 10 BOLD runs found per subject (across all tasks and sessions), the following preprocessing was performed. First, a reference volume and its skull-stripped version were generated using a custom methodology of *fMRIPrep*. A deformation field to correct for susceptibility distortions was estimated based on two echo-planar imaging (EPI) references with opposing phase-encoding directions, using 3dQwarp Cox and Hyde (1997) (AFNI 20160207). Based on the estimated susceptibility distortion, an unwarped BOLD reference was calculated for a more accurate co-registration with the anatomical reference. The BOLD reference was then co-registered to the T1w reference using bbregister (FreeSurfer) which implements boundary-based registration (Greve and Fischl 2009). Co-registration was configured with six degrees of freedom. Head-motion parameters with respect to the BOLD reference (transformation matrices, and six corresponding rotation and translation parameters) are estimated before any spatiotemporal filtering using mcflirt (FSL 5.0.9, Jenkinson et al. 2002). The BOLD time-series, were resampled to surfaces on the following spaces: *fsaverage5*. The BOLD time-series (including slice-timing correction when applied) were resampled onto their original, native space by applying a single, composite transform to correct for head-motion and susceptibility distortions. These resampled BOLD time-series will be referred to as *preprocessed BOLD in original space*, or just *preprocessed BOLD*. The BOLD time-series were resampled into standard space, generating a *preprocessed BOLD run in ['MNI152NLin6Asym'] space*. First, a reference volume and its skull-stripped version were generated using a custom methodology of *fMRIPrep*. Automatic removal of motion artifacts using independent component analysis (ICA-AROMA, Pruim et al. 2015) was performed on the *preprocessed BOLD on MNI space* time-series after removal of non-steady state volumes and spatial smoothing with an isotropic, Gaussian kernel of 6mm FWHM (full-width half-maximum). Corresponding "non-aggresively" denoised runs were produced after such smoothing. Additionally, the "aggressive" noise-regressors were collected and placed in the corresponding confounds file[1]. Several confounding time-series were calculated based on the *preprocessed BOLD*: framewise displacement (FD), DVARS and three region-wise global signals. FD and DVARS are calculated for each functional run, both using their implementations in *Nipype* (following the definitions by Power et al. 2014). The three global signals are extracted within the CSF, the WM, and the whole-brain masks. Additionally, a set of physiological regressors were extracted to allow for component-based noise correction (*CompCor*, Behzadi et al. 2007). Principal components are estimated after high-pass filtering the *preprocessed BOLD* time-series (using a discrete cosine filter with 128s cut-off) for the two *CompCor* variants: temporal (tCompCor) and anatomical (aCompCor). tCompCor components are then calculated from the top 5% variable

[1] *Aggressive denoising* fully regresses out independent component maps identified as noise, removing all overlapping variance, including variance shared with signal components. *Non-aggressive denoising* regresses out component maps to remove variance from only the noise components i.e., motion-related components that overlap with signal remain in the data).

© 2025 Hidalgo-Lopez E et al. *JAMA Network Open*.

voxels within a mask covering the subcortical regions. This subcortical mask is obtained by heavily eroding the brain mask, which ensures it does not include cortical GM regions. For aCompCor, components are calculated within the intersection of the aforementioned mask and the union of CSF and WM masks calculated in T1w space, after their projection to the native space of each functional run (using the inverse BOLD-to-T1w transformation). Components are also calculated separately within the WM and CSF masks. For each CompCor decomposition, the *k* components with the largest singular values are retained, such that the retained components' time series are sufficient to explain 50 percent of variance across the nuisance mask (CSF, WM, combined, or temporal). The remaining components are dropped from consideration. The head-motion estimates calculated in the correction step were also placed within the corresponding confounds file. The confound time series derived from head motion estimates and global signals were expanded with the inclusion of temporal derivatives and quadratic terms for each (Satterthwaite et al. 2013). Frames that exceeded a threshold of 0.5 mm FD or 1.5 standardised DVARS were annotated as motion outliers. All resamplings can be performed with *a single interpolation step* by composing all the pertinent transformations (i.e. head-motion transform matrices, susceptibility distortion correction when available, and co-registrations to anatomical and output spaces). Gridded (volumetric) resamplings were performed using antsApplyTransforms (ANTs), configured with Lanczos interpolation to minimize the smoothing effects of other kernels (Lanczos 1964). Non-gridded (surface) resamplings were performed using mri_vol2surf (FreeSurfer).

Many internal operations of *fMRIPrep* use *Nilearn* 0.5.2 (Abraham et al. 2014, RRID:SCR_001362), mostly within the functional processing workflow. For more details of the pipeline, see [the section corresponding to workflows in *fMRIPrep*'s documentation].

References fMRIPrep

Abraham, Alexandre, Fabian Pedregosa, Michael Eickenberg, Philippe Gervais, Andreas Mueller, Jean Kossaifi, Alexandre Gramfort, Bertrand Thirion, and Gael Varoquaux. 2014. "Machine Learning for Neuroimaging with Scikit-Learn." *Frontiers in Neuroinformatics* 8. https://doi.org/10.3389/fninf.2014.00014.

Avants, B.B., C.L. Epstein, M. Grossman, and J.C. Gee. 2008. "Symmetric Diffeomorphic Image Registration with Cross-Correlation: Evaluating Automated Labeling of Elderly and Neurodegenerative Brain." *Medical Image Analysis* 12 (1): 26–41. https://doi.org/10.1016/j.media.2007.06.004.

Behzadi, Yashar, Khaled Restom, Joy Liau, and Thomas T. Liu. 2007. "A Component Based Noise Correction Method (CompCor) for BOLD and Perfusion Based fMRI." *NeuroImage* 37 (1): 90–101. https://doi.org/10.1016/j.neuroimage.2007.04.042.

Cox, Robert W., and James S. Hyde. 1997. "Software Tools for Analysis and Visualization of fMRI Data." *NMR in Biomedicine* 10 (4-5): 171–78. https://doi.org/10.1002/(SICI)1099-1492(199706/08)10:4/5<171::AID-NBM453>3.0.CO;2-L.

Dale, Anders M., Bruce Fischl, and Martin I. Sereno. 1999. "Cortical Surface-Based Analysis: I. Segmentation and Surface Reconstruction." *NeuroImage* 9 (2): 179–94. https://doi.org/10.1006/nimg.1998.0395.

Esteban, Oscar, Ross Blair, Christopher J. Markiewicz, Shoshana L. Berleant, Craig Moodie, Feilong Ma, Ayse Ilkay Isik, et al. 2018. "FMRIPrep." *Software*. Zenodo. https://doi.org/10.5281/zenodo.852659.

Esteban, Oscar, Christopher Markiewicz, Ross W Blair, Craig Moodie, Ayse Ilkay Isik, Asier Erramuzpe Aliaga, James Kent, et al. 2018. "fMRIPrep: A Robust Preprocessing Pipeline for Functional MRI." *Nature Methods*. https://doi.org/10.1038/s41592-018-0235-4.

Evans, AC, AL Janke, DL Collins, and S Baillet. 2012. "Brain Templates and Atlases." *NeuroImage* 62 (2): 911–22. https://doi.org/10.1016/j.neuroimage.2012.01.024.

Gorgolewski, K., C. D. Burns, C. Madison, D. Clark, Y. O. Halchenko, M. L. Waskom, and S. Ghosh. 2011. "Nipype: A Flexible, Lightweight and Extensible Neuroimaging Data Processing Framework in Python." *Frontiers in Neuroinformatics* 5: 13. https://doi.org/10.3389/fninf.2011.00013.

Gorgolewski, Krzysztof J., Oscar Esteban, Christopher J. Markiewicz, Erik Ziegler, David Gage Ellis, Michael Philipp Notter, Dorota Jarecka, et al. 2018. "Nipype." *Software*. Zenodo. https://doi.org/10.5281/zenodo.596855.

Greve, Douglas N, and Bruce Fischl. 2009. "Accurate and Robust Brain Image Alignment Using Boundary-Based Registration." *NeuroImage* 48 (1): 63–72. https://doi.org/10.1016/j.neuroimage.2009.06.060.

Jenkinson, Mark, Peter Bannister, Michael Brady, and Stephen Smith. 2002. "Improved Optimization for the Robust and Accurate Linear Registration and Motion Correction of Brain Images." *NeuroImage* 17 (2): 825–41. https://doi.org/10.1006/nimg.2002.1132.

© 2025 Hidalgo-Lopez E et al. *JAMA Network Open*.

Klein, Arno, Satrajit S. Ghosh, Forrest S. Bao, Joachim Giard, Yrjö Häme, Eliezer Stavsky, Noah Lee, et al. 2017. "Mindboggling Morphometry of Human Brains." *PLOS Computational Biology* 13 (2): e1005350. https://doi.org/10.1371/journal.pcbi.1005350.

Lanczos, C. 1964. "Evaluation of Noisy Data." *Journal of the Society for Industrial and Applied Mathematics Series B Numerical Analysis* 1 (1): 76–85. https://doi.org/10.1137/0701007.

Power, Jonathan D., Anish Mitra, Timothy O. Laumann, Abraham Z. Snyder, Bradley L. Schlaggar, and Steven E. Petersen. 2014. "Methods to Detect, Characterize, and Remove Motion Artifact in Resting State fMRI." *NeuroImage* 84 (Supplement C): 320–41. https://doi.org/10.1016/j.neuroimage.2013.08.048.

Pruim, Raimon H. R., Maarten Mennes, Daan van Rooij, Alberto Llera, Jan K. Buitelaar, and Christian F. Beckmann. 2015. "ICA-AROMA: A Robust ICA-Based Strategy for Removing Motion Artifacts from fMRI Data." *NeuroImage* 112 (Supplement C): 267–77. https://doi.org/10.1016/j.neuroimage.2015.02.064.

Satterthwaite, Theodore D., Mark A. Elliott, Raphael T. Gerraty, Kosha Ruparel, James Loughead, Monica E. Calkins, Simon B. Eickhoff, et al. 2013. "An improved framework for confound regression and filtering for control of motion artifact in the preprocessing of resting-state functional connectivity data." *NeuroImage* 64 (1): 240–56. https://doi.org/10.1016/j.neuroimage.2012.08.052.

Tustison, N. J., B. B. Avants, P. A. Cook, Y. Zheng, A. Egan, P. A. Yushkevich, and J. C. Gee. 2010. "N4ITK: Improved N3 Bias Correction." *IEEE Transactions on Medical Imaging* 29 (6): 1310–20. https://doi.org/10.1109/TMI.2010.2046908.

Zhang, Y., M. Brady, and S. Smith. 2001. "Segmentation of Brain MR Images Through a Hidden Markov Random Field Model and the Expectation-Maximization Algorithm." *IEEE Transactions on Medical Imaging* 20 (1): 45–57. https://doi.org/10.1109/42.906424.

Copyright Waiver

The above boilerplate text was automatically generated by fMRIPrep with the express intention that users should copy and paste this text into their manuscripts *unchanged*. It is released under the CC0 license.

Time series extraction

Volumes exceeding a framewise displacement threshold of 0.5mm were marked to be censored. Covariates were regressed out of the time series in a single step, including: linear trend, 24 motion parameters (original translations/rotations + derivatives + quadratics), aCompCor with 5 cerebrospinal fluid and 5 white matter principal components and ICA-AROMA aggressive components, high pass filtering at 0.008Hz, and censored volumes.

ROI Selection

eTable 1 shows a summary of the ROIs with atlas nomenclature and justification based on the literature.

Five ROIs define the SLN: bilateral mid insula, left dorsal anterior cingulate cortex, and bilateral dorsolateral prefrontal cortices. Areas corresponding to the mid insula parcels have previously evidenced altered resting-state connectivity in children who develop multisite pain[10] and adults with fibromyalgia.[11] Specifically in children, increased functional connectivity with sensorimotor areas has been reported in juvenile fibromyalgia[12] and as a predictor of multisite pain.[10] Medial regions, such as the left dorsal anterior cingulate cortex, exhibit alterations in diverse pain conditions, such as reduced grey matter volume in children with irritable bowel syndrome[13] and juvenile fibromyalgia,[14] and differential functional connectivity in adult fibromyalgia.[15] Finally, bilateral dorso-lateral prefrontal cortex regions have been suggested as crucial for pain regulation, with different activation between adult chronic pain patients and controls (see review[16]). Moreover, in children with irritable bowel syndrome, cortical thinning has been found in the left hemisphere,[13] and its activity predicts higher pain intensity in some circumstances.[17]

Five ROIs define the SMN: bilateral regions containing lateral S1/M1, a medial S1/M1 region, and posterior insula. Stronger functional connectivity of lateral S1/M1 with the SLN has been seen in adults with chronic widespread pain[18] and suggested as a chronic pain precursor in children.[10] Next, medial sensorimotor areas show decreased functional connectivity in juvenile fibromyalgia.[12] Last, the dorsal posterior insula has been suggested as a key therapeutic target for different pain conditions.[19] Specifically in children, right posterior insular activity has been shown to predict higher pain intensity,[17] and its connectivity with M1 to precede multisite pain.[10]

Four ROIs define the DMN: right ventromedial prefrontal cortex (vmPFC), right precuneus, and bilateral inferior parietal lobe (IPL). Diminished oscillatory activity in the right vmPFC has been shown to predict multisite pain in children,[10] and its functional connectivity with the nucleus accumbens to predict pain chronification.[20] In girls with irritable bowel syndrome, lower connectivity between right precuneus and the bilateral medial cingulate cortex has been reported.[13] These patients also showed a differential IPL structure and connectivity, with cortical thickness relating to the severity of abdominal pain complaints,[21] and greater connectivity with the left thalamus being observed.[13]

eTable 1. Definitions of Regions of Interest Used in Functional Connectivity Analyses According to Their Schaefer Atlas Nomenclature

	LEFT HEMISPHERE	RIGHT HEMISPHERE
**Salience network**		
Mid insula	*17Networks_LH_SalVentAttnA_Ins_1* [10,22,23]	*17Networks_RH_SalVentAttnA_Ins_1* [12,14,22]
Dorsal anterior cingulate cortex	*17Networks_LH_SalVentAttnB_PFCmp_1* [13,14,22-24]	
Dorsolateral prefrontal cortex	*17Networks_LH_SalVentAttnB_PFCl_1* [13,17,22]	*17Networks_RH_SalVentAttnB_PFCl_1* [22]
**Somatosensory and motor network**		
Lateral somatosensory and motor cortex	*17Networks_LH_SomMotA_6* [10,22]	*17Networks_RH_SomMotA_7* [10,22]
Medial somatosensory and motor cortex	*17Networks_LH_SomMotA_12* [12]	
Posterior insula	*17Networks_LH_SomMotB_S2_2* [19]	*17Networks_RH_SomMotB_S2_2* [10,17]
**Default mode network**		
Ventromedial prefrontal cortex		*17Networks_RH_DefaultA_PFCm_1* [10,22,24,25]
Precuneus/posterior cingulate cortex		*17Networks_RH_DefaultA_pCunPCC_1* [13,22,24,25]
Inferior parietal lobe	*17Networks_LH_DefaultA_IPL_1* [13,21,22,24,25]	*17Networks_RH_DefaultA_IPL_2* [22,24,25]

© 2025 Hidalgo-Lopez E et al. *JAMA Network Open*.

eTable 2. Matched Sample Demographics, Showing Percentages by Sex and Subgroups

	Sex		Subgroups			df	F or X^{2*}	p-value
			No pain	Regional	Multisite			
**Pubertal status (mean and SD)**	*male*		1.96 (0.56)	1.91 (0.54)	1.96 (0.56)	2, 1005	0.87	0.42
	female		2.51 (0.63)	2.52 (0.61)	2.51 (0.64)	2, 1041	0.10	0.91
**Handedness (%)**	*male*	*right*	81.25	80.06	78.57	4	2.44	0.65
		left	5.95	4.46	6.55			
		ambi	12.80	15.48	14.88			
	female	*right*	79.89	81.90	79.60	4	3.71	0.45
		left	2.87	4.60	4.89			
		ambi	17.24	13.51	15.52			
**Race/ethnicity (%)**	*male*	*White*	66.96	68.45	66.07	8	1.10	1.00
		Black	5.95	6.55	5.95			
		Hispanic	17.56	16.37	17.86			
		Asian	0.60	0.60	0.89			
		Other	8.93	8.04	9.23			
	female	*White*	58.05	62.64	60.34	8	5.41	0.71
		Black	9.77	7.18	8.05			
		Hispanic	17.53	18.39	17.82			
		Asian	1.44	2.30	1.44			
		Other	13.22	9.48	12.36			

Chi-squared or ANOVA tests were used when percentages or mean and SD were indicated, respectively.

eFigure 1. Schematic Summary of the Analytical Design, Including a Flow Chart Describing GIMME, Its Model Fitting Procedures, and Graphical Depiction of Present Results

Each person-specific network is characterized by individual indexes presented at the bottom of the figure: a) complexity, or the number of connections in the entire network, b) within-network densities, or the number of connections between two nodes of the same network (e.g., SMN), relative to complexity, and c) between-network densities, or number of connections between two nodes of different networks (e.g., SMN-SLN), relative to complexity.

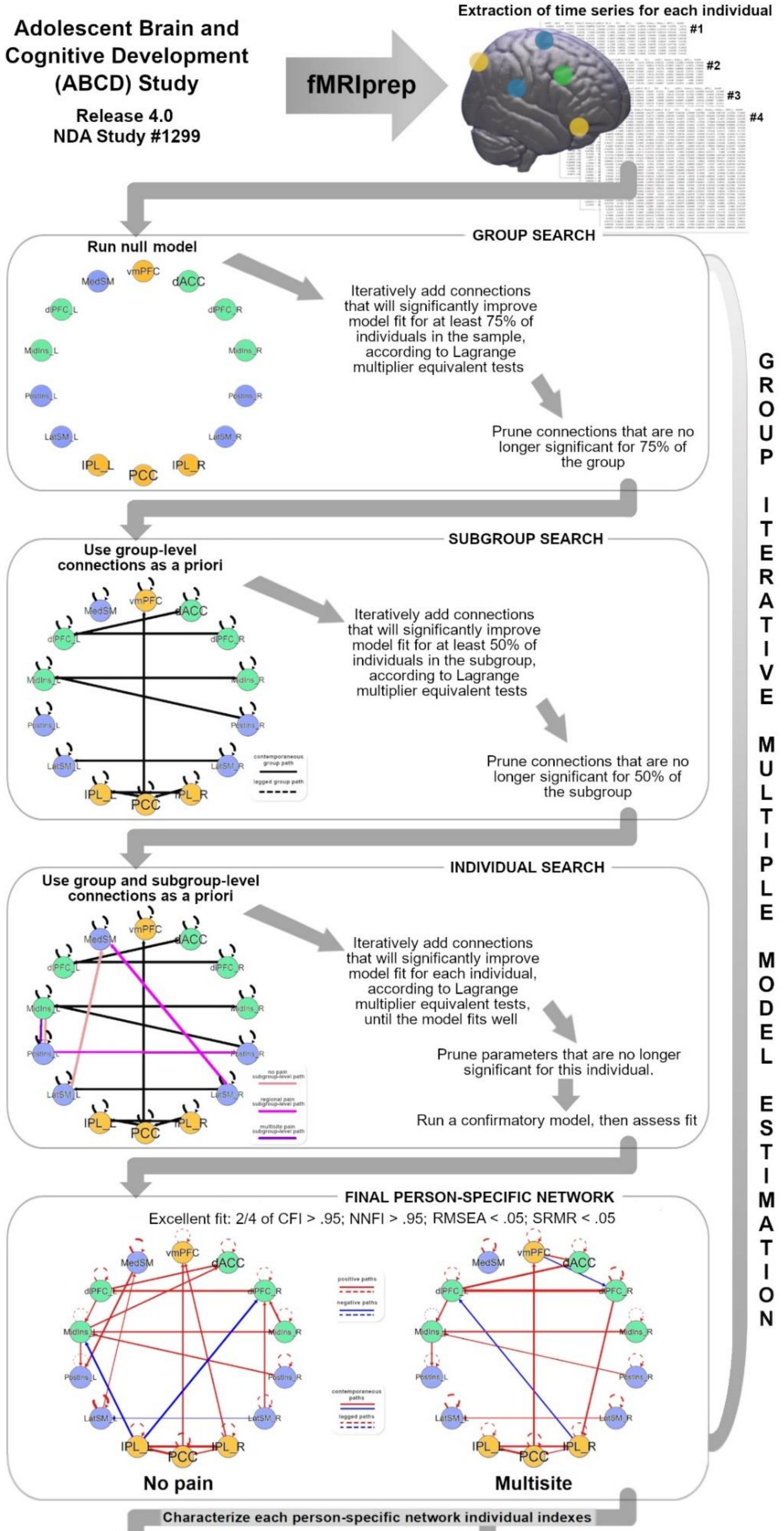

Complexity	33
SMN relative	0.24
SLN relative	0.33
DMN relative	0.27
SLN-SMN relative	0.09
DMN-SLN relative	0.06
DMN-SMN relative	0.00

Complexity	29
SMN relative	0.21
SLN relative	0.31
DMN relative	0.28
SLN-SMN relative	0.07
DMN-SLN relative	0.14
DMN-SMN relative	0.00

© 2025 Hidalgo-Lopez E et al. *JAMA Network Open*.

eTable 3. Descriptive Statistics for Network Densities and Symptom Domains by Sex and Subgroups (No Pain, Regional Pain, and Multisite Pain)

Brain connectivity (n=2052)						
	**Males**			**Females**		
	**No Pain**	**Regional**	**Multisite**	**No Pain**	**Regional**	**Multisite**
	Mean (SD)	*Mean (SD)*	*Mean (SD)*	*Mean (SD)*	*Mean (SD)*	*Mean (SD)*
Relative density of SMN connectivity	.277 (.034)	.292 (.033)	.263 (.035)	.291 (.030)	.28 (.032)	.281 (.033)
Relative density of DMN connectivity	.262 (.028)	.259 (.026)	.266 (.029)	.258 (.025)	.262 (.026)	.264 (.028)
Relative density of SLN connectivity	.290 (.030)	.286 (.030)	.292 (.031)	.283 (.028)	.287 (.029)	.288 (.029)
Relative density between SMN-SLN connectivity	.094 (.030)	.079 (.034)	.093 (.030)	.091 (.030)	.091 (.031)	.091 (.030)
Relative density between DMN-SLN connectivity	.057 (.038)	.059 (.038)	.06 (.039)	.059 (.039)	.061 (.040)	.057 (.037)
Relative density between DMN-SMN connectivity	.021 (.025)	.024 (.029)	.026 (.027)	.017 (.022)	.020 (.023)	.019 (.024)

Brief Problem Monitor						
	**Males**			**Females**		
	**No Pain**	**Regional**	**Multisite**	**No Pain**	**Regional**	**Multisite**
	Mean (SD)	*Mean (SD)*	*Mean (SD)*	*Mean (SD)*	*Mean (SD)*	*Mean (SD)*
BPM Attention Problems Score (n=1924)	2.96 (2.56)	3.35 (2.56)	4.16 (2.82)	3.04 (2.75)	3.42 (2.73)	3.94 (2.82)
BPM Internalizing Problems Score (n=2001)	1.14 (1.58)	1.77 (2.13)	2.28 (2.47)	2.01 (2.46)	2.40 (2.53)	3.21 (2.85)
BPM Externalizing Problems Score (n=1970)	1.84 (1.80)	2.10 (1.83)	2.79 (2.32)	1.96 (1.94)	2.20 (1.99)	2.74 (2.25)
BPM Total Problems Score (n=1869)	5.84 (4.95)	7.19 (5.06)	9.13 (6.15)	6.91 (5.80)	7.96 (5.80)	9.82 (6.31)

Sleep Disturbances Scale for Children (n=2049)						
	**Males**			**Females**		
	**No Pain**	**Regional**	**Multisite**	**No Pain**	**Regional**	**Multisite**
	Mean (SD)	*Mean (SD)*	*Mean (SD)*	*Mean (SD)*	*Mean (SD)*	*Mean (SD)*
SDSC Disorders of Initiating and Maintaining Sleep	12.08 (3.73)	12.14 (3.92)	12.51 (3.77)	11.94 (3.74)	11.92 (3.42)	13.04 (3.81)
SDSC Sleep Breathing Disorders	3.64 (1.12)	3.71 (1.22)	3.77 (1.36)	3.57 (0.92)	3.60 (1.09)	3.59 (0.93)
SDSC Disorders of Arousal	3.40 (0.87)	3.33 (0.76)	3.41 (0.80)	3.24 (0.58)	3.30 (0.64)	3.27 (0.64)
SDSC Sleep-Wake Transition Disorders	8.11 (2.60)	7.87 (2.61)	8.39 (2.73)	7.52 (1.99)	7.90 (2.22)	8.04 (2.58)
SDSC Disorders of Excessive Somnolence	7.21 (2.70)	7.39 (2.71)	7.38 (2.59)	7.09 (2.45)	7.22 (2.37)	7.80 (2.60)
SDSC Sleep Hyperhidrosis	2.39 (0.99)	2.39 (0.98)	2.50 (1.17)	2.21 (0.76)	2.24 (0.68)	2.31 (0.99)
SDSC Total Score	36.84 (8.03)	36.82 (8.52)	37.96 (8.40)	35.57 (7.38)	36.18 (6.93)	38.03 (7.66)

eTable 4. GIMME Model Fit Indices Averaged by Sex and Subgroups

© 2025 Hidalgo-Lopez E et al. *JAMA Network Open*.

Males			
Model fit indices	**No pain**	**Regional**	**Multisite**
**X^2**	399.88	401.26	398.02
df	253.97	253.72	254.48
**CFI**	0.92	0.93	0.92
**NNFI**	0.89	0.89	0.89
**RMSEA**	0.04	0.04	0.04
**SRMR**	0.05	0.05	0.05
**Females**			
Model fit indices	**No pain**	**Regional**	**Multisite**
**X^2**	402.06	400.51	397.39
df	253.76	254.03	254.37
**CFI**	0.92	0.93	0.93
**NNFI**	0.89	0.89	0.89
**RMSEA**	0.04	0.04	0.04
**SRMR**	0.05	0.05	0.05

Each model is accepted to have excellent fit according to two out of four commonly accepted fit indices: comparative fit index (CFI) ⩾ .95; non-normed fit index (NNFI; also known as the Tucker-Lewis index) ⩾ .95; root mean squared error of approximation (RMSEA) ⩽ .05; standardized root mean residual (SRMR) ⩽ .05.

© 2025 Hidalgo-Lopez E et al. *JAMA Network Open*.

eFigure 2. CS-GIMME Models for Each Subgroup by Sex, With Examples of Person-Specific Maps

Summary maps, depicting results across males (a) and females (c) show group-level (thick black lines), subgroup-level (thick pink lines), and individual-level (thin grey lines) connections. Continuous lines represent contemporaneous connections, whereas dashed lines represent lagged connections. From the 23 group-level connections for males and 22 for females, 14 were autoregressions (estimated a priori, dashed circular lines), and 9 or 8, respectively, were contemporaneous connections. Four of these contemporaneous paths corresponded to contralateral connections between homologous regions, as expected. Three illustrative person-specific maps for each sex, one per subgroup, are shown for males (b) and females (d). Red lines represent positive connections, whereas blue lines represent negative connections. All connections are directional with line thickness corresponding to connection magnitude.

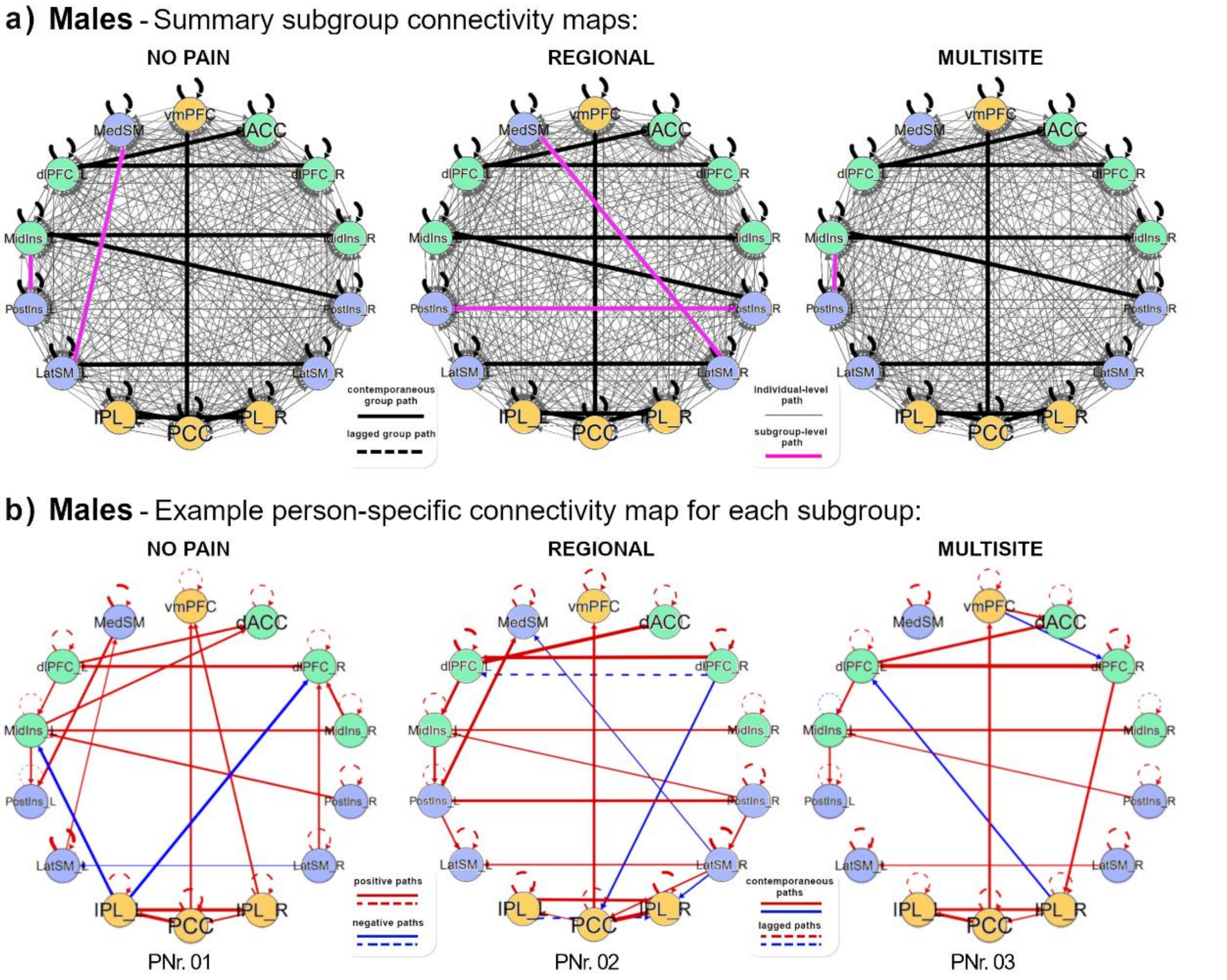

© 2025 Hidalgo-Lopez E et al. *JAMA Network Open*.

c) Females - Summary subgroup connectivity maps:

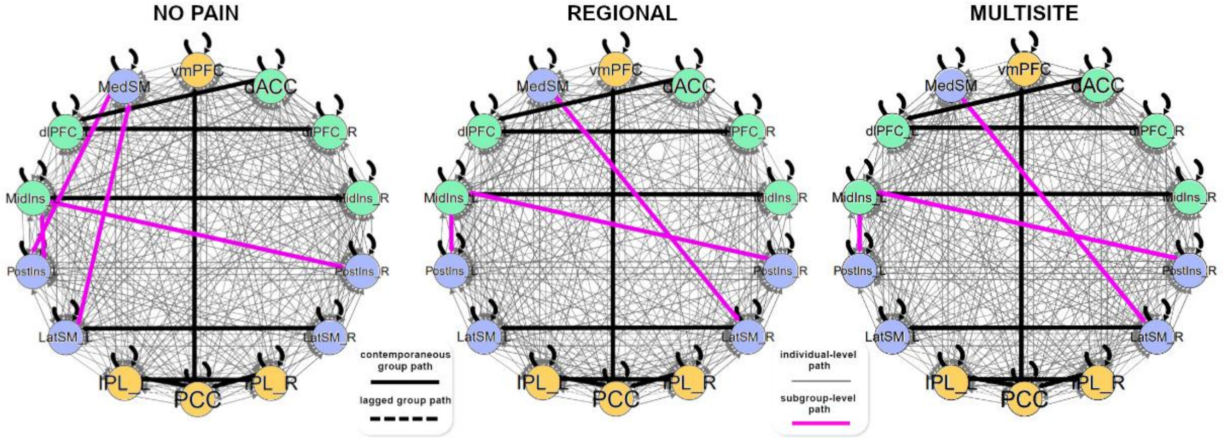

d) Females - Example person-specific connectivity map for each subgroup:

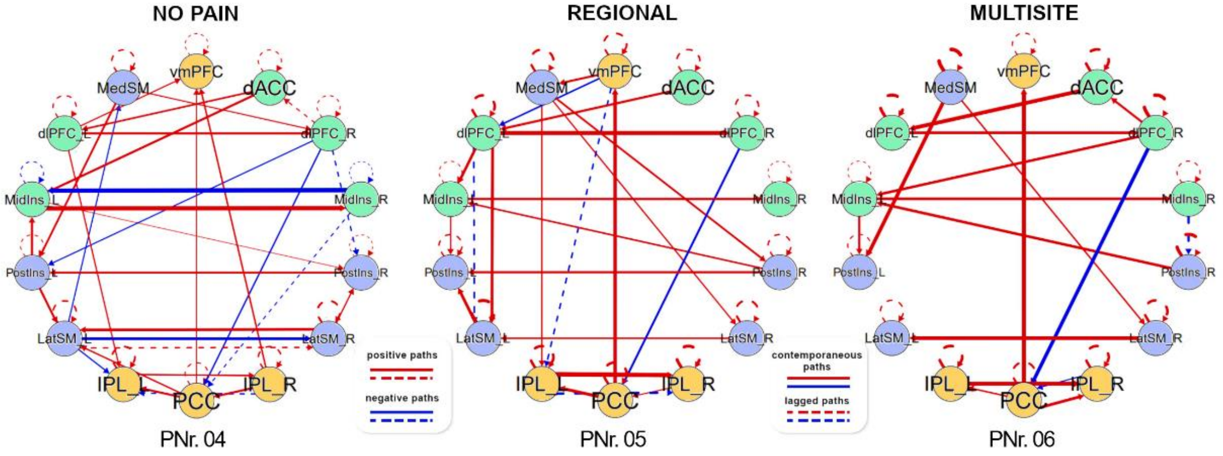

© 2025 Hidalgo-Lopez E et al. *JAMA Network Open*.

eFigure 3. Differences in Network Connectivity Densities Between No Pain, Regional Pain, and Multisite Pain Subgroups Stratified by Sex

Differences in (a) males, and (b) females in within-network densities (in yellow-orange) and between-network densities (in red). Red lines with asterisks showed pair-wise Tukey HSD comparisons significant at: *p<0.05, **p<0.01, and ***p<0.001.

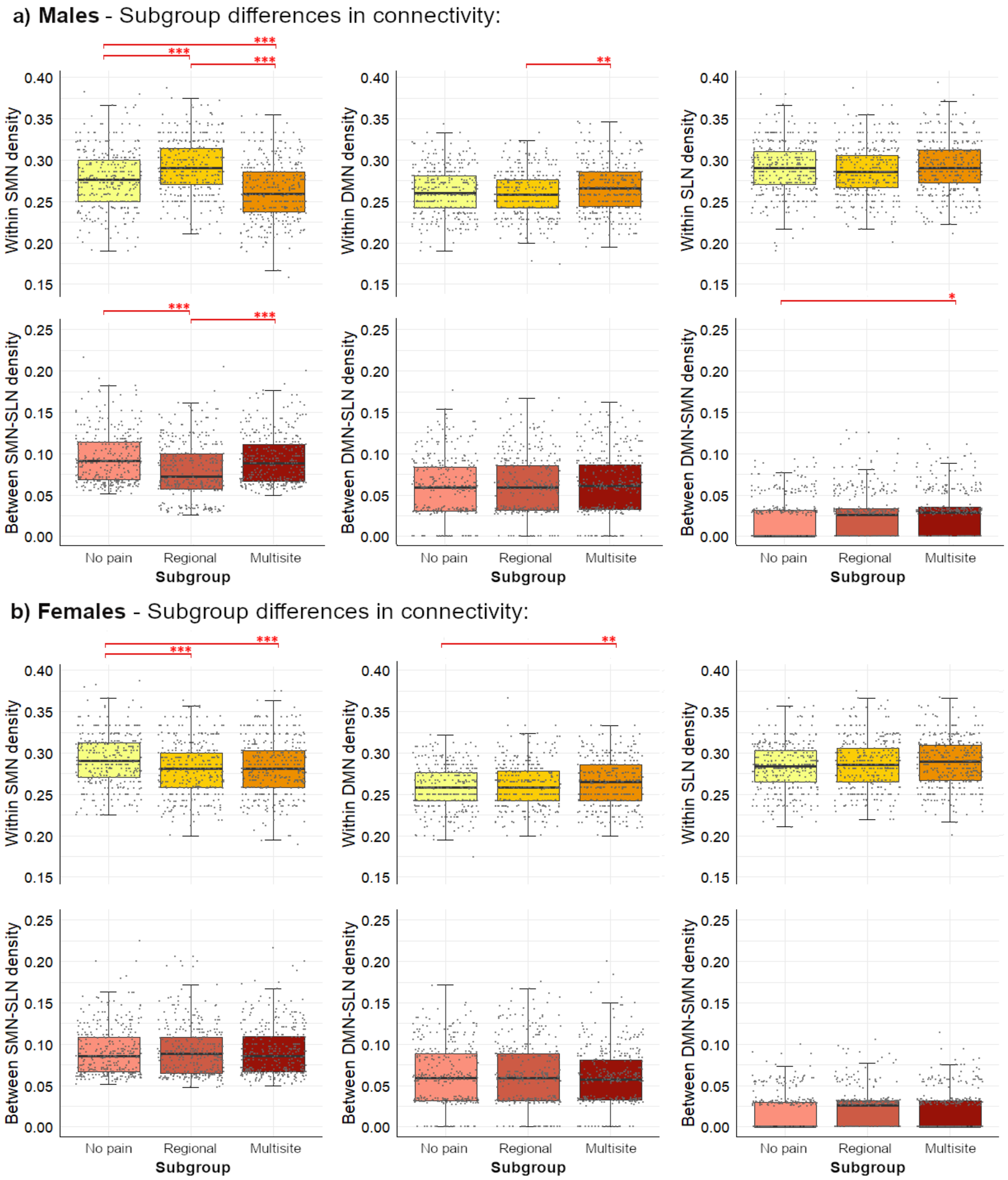

© 2025 Hidalgo-Lopez E et al. *JAMA Network Open*.

eTable 5. One-Way Analysis of Variance Results for Network Densities and Symptom Domains (Behavior Problems and Sleep Disturbances)

**Brain connectivity (n=2052)**								
	**Males**				**Females**			
	$F_{(2, 1005)}$	η^2	*p*	p_{FDR}	$F_{(2, 1041)}$	η^2	*p*	p_{FDR}
Relative density of SMN connectivity	61.40	.109	<.001	**<.001**	13.38	.025	<.001	**<.001**
Relative density of DMN connectivity	5.88	.012	.003	**.005**	4.22	.008	.015	**.023**
Relative density of SLN connectivity	2.56	.005	.078	.078	2.34	.004	.097	.097
Relative density between SMN-SLN connectivity	22.31	.043	<.001	**<.001**	.001	.000	.999	.999
Relative density between DMN-SLN connectivity	.710	.001	.492	.492	.983	.002	.375	.563
Relative density between DMN-SMN connectivity	3.55	.007	.029	**.044**	1.13	.002	.324	.563
**Brief Problem Monitor**								
	**Males**				**Females**			
	$F_{(2, n-3)}$	η^2	*p*	p_{FDR}	$F_{(2, n-3)}$	η^2	*p*	p_{FDR}
BPM Attention Problems Score (n=1924)	16.62	.034	<.001	**<.001**	8.50	.017	<.001	**<.001**
BPM Internalizing Problems Score (n=2001)	24.62	.048	<.001	**<.001**	18.41	.035	<.001	**<.001**
BPM Externalizing Problems Score (n=1970)	19.20	.038	<.001	**<.001**	12.77	.025	<.001	**<.001**
BPM Total Problems Score (n=1869)	28.12	.058	<.001	**<.001**	19.12	.039	<.001	**<.001**
**Sleep Disturbances Scale for Children (n=2049)**								
	**Males**				**Females**			
	$F_{(2, 1004)}$	η^2	*p*	p_{FDR}	$F_{(2, 1039)}$	η^2	*p*	p_{FDR}
SDSC Disorders of Initiating and Maintaining Sleep	1.28	.003	.278	.481	10.62	.020	<.001	**.002**
SDSC Sleep Breathing Disorders	.890	.002	.412	.481	.099	.000	.906	.906
SDSC Disorders of Arousal	1.06	.002	.346	.481	.808	.002	.446	.521
SDSC Sleep-Wake Transition Disorders	3.31	.007	.037	.259	4.78	.009	<.010	**.016**
SDSC Disorders of Excessive Somnolence	.440	.001	.643	.643	8.03	.015	<.001	**.002**
SDSC Sleep Hyperhidrosis	1.19	.002	.304	.481	1.29	.002	.277	.388
SDSC Total Score	2.09	.004	.124	.434	10.64	.020	<.001	**.002**

Both males and females showed between subgroup differences in sensorimotor network (SMN) and default mode network (DMN) densities. Only males showed differences in between SMN and salience (SLN), and between DMN and SMN densities. Both males and females in the multisite subgroup showed increased behavior problem scores and its subscales of Internalizing, Externalizing, and Attention problems compared to the no pain and regional subgroups. Only in females, the multisite subgroup had higher total sleep disturbances and subscales of Disorders of Initiating and Maintaining Sleep, Sleep-Wake Transition Disorders, and Excessive Somnolence, compared to the no pain and regional subgroups. Post Hoc Tukey's HSD tests results are shown in Figure 3 of the main text.

eTable 6. Number of Participants Scanned by Each MRI Manufacturer by Sex and Subgroups (No Pain, Regional Pain, and Multisite Pain)

	**Males**			**Females**		
	**No Pain**	**Regional**	**Multisite**	**No Pain**	**Regional**	**Multisite**
GE	95	95	108	100	104	114
Philips	24	32	28	24	32	33
Siemens	217	209	200	224	212	201

First, we corroborated that there were no differences in the scanner manufacturer distribution between subgroups. For males $\chi2$ =2.97, df=8, p=0.56. For females $\chi2$ =3.87, df=8, p=0.42. Second, we peformed linear mixed models, testing the differences between subgroups on neural network densities, while adding scanner manufacturer as random effects (*SMN_relative ~ Subgroup + (1 |mri_info_manufacturer).* Findings were consistent with ANOVA results (compare eTable 5 with eTable 7).

© 2025 Hidalgo-Lopez E et al. *JAMA Network Open*.

eTable 7. Linear Mixed Models Results for Subgroup Differences in Network Densities With Scanner Manufacturer (3 Groups) as Random Effects

**MALES**	**SMN**		**DMN**		**SLN**		**SLN-SMN**		**DMN-SLN**		**DMN-SMN**	
Predictors	*Statistic*	*p*	*Statistic*	*p*	*Statistic*	*p*	*Statistic*	*p*	*Statistic*	*p*	*Statistic*	*p*
Subgroups	61.40	**<0.001***	5.78	**0.005***	2.58	0.08	22.50	**<0.001***	0.91	0.40	3.55	**0.029***
Random Effects												
Variance	<0.001		<0.001		<0.001		<0.001		<0.001		<0.001	
**FEMALES**	**SMN**		**DMN**		**SLN**		**SLN-SMN**		**DMN-SLN**		**DMN-SMN**	
Predictors	*Statistic*	*p*	*Statistic*	*p*	*Statistic*	*p*	*Statistic*	*p*	*Statistic*	*p*	*Statistic*	*p*
Subgroups	13.38	**<0.001***	4.04	**0.018***	2.17	0.11	0	0.99	0.87	0.41	1.13	0.32
Random Effects												
Variance	<0.001		<0.001		<0.001		<0.001		<0.001		<0.001	

Findings concerning between-subgroup differences in brain connectivity remained consistent with those reported in eTable 5.
*Survive pFDR corrections threshold.

eTable 8. Descriptive Statistics for Movement Parameters by Sex and Subgroups (No Pain, Regional Pain, and Multisite Pain)

	**Males**			**Females**		
	**No Pain**	**Regional**	**Multisite**	**No Pain**	**Regional**	**Multisite**
	Mean (SD)	*Mean (SD)*	*Mean (SD)*	*Mean (SD)*	*Mean (SD)*	*Mean (SD)*
Mean framewise displacement	0.19 (0.07)	0.18 (0.07)	0.18 (0.07)	0.17 (0.07)	0.17 (0.07)	0.17 (0.07)
Mean number of censored points	14.80 (16.76)	12.26 (14.93)	13.59 (15.42)	10.64 (14.14)	9.57 (12.40)	10.59 (14.41)

Then, we corroborated that there were no differences in movement parameters (mean framewise displacement and the number of censored timepoints) between subgroups. Finally, we tested the differences between subgroups on neural network densities, while controlling for movement parameters, through ANCOVAs (*brain connectivity ~ subgroup + movement parameter*). Findings were consistent with ANOVA results (compare eTable 5 with eTable 10).

eTable 9. One-Way Analysis of Variance Results for Differences Between Subgroups in Movement Parameters

	**Males**		**Females**	
	$F_{(2, 1005)}$	*p*	$F_{(2, 1041)}$	*p*
Mean framewise displacement	1.42	0.24	0.47	0.63
Mean number of censored timepoints	2.19	0.11	0.68	0.51

No differences between the three subgroups were found.

© 2025 Hidalgo-Lopez E et al. *JAMA Network Open*.

eFigure 4. Differences in Behavioral Problems Among No Pain, Regional Pain, and Multisite Pain Subgroups by Sex

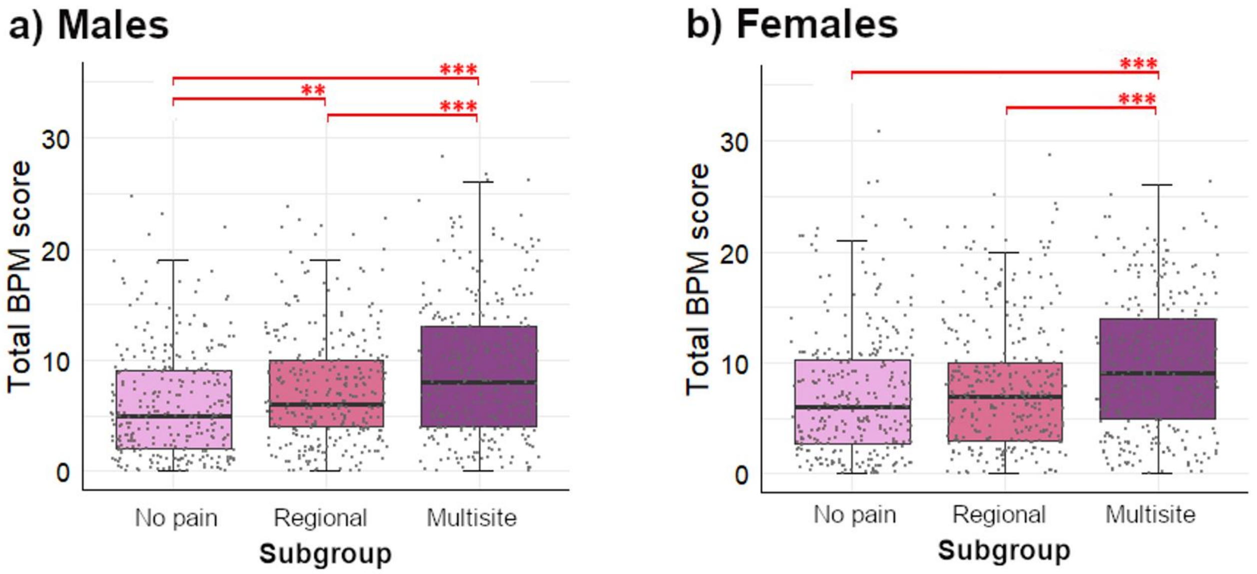

Differences in males (a) and females (b) in behavior problems (purple) are shown. Both males and females with pain had heightened behavior problem. Red lines with asterisks show pair-wise Tukey HSD comparisons significant at: *p<0.05, **p<0.01, and ***p<0.001.

© 2025 Hidalgo-Lopez E et al. *JAMA Network Open*.

eTable 10. Analysis of Covariance Results for Subgroup Differences in Network Densities With Movement Parameters as Covariates

	**Subgroup**												**Mean framewise displacement**					
	**Males**						**Females**						**Males**			**Females**		
	$F_{(2, 1004)}$	p	p_{FDR}	p_{tukey} R vs M	p_{tukey} R vs NP	p_{tukey} M vs NP	$F_{(2, 1040)}$	p	p_{FDR}	p_{tukey} R vs M	p_{tukey} R vs NP	p_{tukey} M vs NP	$F_{(1, 1004)}$	p	p_{FDR}	$F_{(1, 1040)}$	p	p_{FDR}
SMN connectivity	61.41	<.001	**<.001**	**<.001**	**<.001**	**<.001**	13.40	<.001	**<.001**	.770	**<.001**	**<.001**	1.18	.280	.280	2.04	.153	.176
DMN connectivity	6.41	.002	**.003**	**<.001**	.130	.140	4.42	.012	**.018**	.310	.200	**.005**	90.80	<.001	**<.001**	50.50	<.001	**<.001**
SLN connectivity	2.59	.080	.080	-	-	-	2.34	.097	.097	-	-	-	15.12	<.001	**<.001**	1.83	.176	.176
SMN-SLN connectivity	23.73	<.001	**<.001**	**<.001**	**<.001**	.940	33.07	.999	.999	-	-	-	64.68	<.001	**<.001**	33.07	<.001	**<.001**
DMN-SLN connectivity	.710	.492	.492	-	-	-	.982	.375	.563	-	-	-	.357	.550	.550	.507	.477	.477
DMN-SMN connectivity	3.65	.026	**.040**	.620	.150	**.010**	1.16	.313	.563	-	-	-	27.80	<.001	**<.001**	30.98	<.001	**<.001**

	**Subgroup**												**Mean number of censored timepoints**					
	**Males**						**Females**						**Males**			**Females**		
	$F_{(2, 1004)}$	p	p_{FDR}	p_{tukey} R vs M	p_{tukey} R vs NP	p_{tukey} M vs NP	$F_{(2, 1040)}$	p	p_{FDR}	p_{tukey} R vs M	p_{tukey} R vs NP	p_{tukey} M vs NP	$F_{(1, 1004)}$	p	p_{FDR}	$F_{(1, 1040)}$	p	p_{FDR}
SMN connectivity	61.70	<.001	**<.001**	**< .001**	**< .001**	**< .001**	13.50	<.001	**<.001**	.730	**< .001**	**< .001**	5.88	.0155	**.016**	9.86	.001	**.002**
DMN connectivity	6.59	.001	**.002**	**<.001**	.080	.170	4.57	.011	**.017**	.250	.330	**.008**	120.59	<.001	**<.001**	85.79	<.001	**<.001**
SLN connectivity	2.60	.075	.075	-	-	-	2.35	.096	.096	-	-	-	12.88	<.001	**<.001**	5.09	.0243	.0243
SMN-SLN connectivity	23.92	<.001	**<.001**	**< .001**	**< .001**	.970	.001	.999	0.999	-	-	-	73.34	<.001	**<.001**	60.7	<.001	**<.001**
DMN-SLN connectivity	.710	.492	.492	-	-	-	.982	.375	0.563	-	-	-	1.50	.221	.221	.028	.867	.867
DMN-SMN connectivity	3.70	.0251	**.037**	.660	.10	**.01**	1.19	.305	0.563	-	-	-	42.74	<.001	**<.001**	55.383	<.001	**<.001**

One-way ANCOVAs were conducted to compare brain connectivity between subgroups, while controlling for movement parameters (mean framewise displacement, top table; and number of censored timepoints, bottom table). Independent of the main effects of the movement parameters on density (shown in the rightmost columns), findings concerning between-subgroup differences in brain connectivity remained consistent with those reported in eTable 5. p_{tukey}: Post Hoc Tukey's HSD tests, NP: no pain, R: regional, M: multisite

© 2025 Hidalgo-Lopez E et al. *JAMA Network Open*.

eTable 11. Correlations Between Network Densities and Symptom Domains

MALES - *Means, standard deviations, and correlations with confidence intervals*

Variable	*M*	*SD*	1	2	3	4	5	6	7
1. BPM Total score	7.36	5.56							
2. SDSC Total score	37.21	8.33	.18** [.12, .24]						
3. Within SMN	0.28	0.04	-.04 [-.11, .02]	-.02 [-.08, .04]					
4. Within DMN	0.26	0.03	-.03 [-.10, .03]	-.01 [-.08, .05]	.05 [-.01, .11]				
5. Within SLN	0.29	0.03	.02 [-.05, .08]	.01 [-.05, .07]	-.09** [-.15, -.03]	.25** [.19, .31]			
6. Between SLN-SMN	0.09	0.03	.01 [-.06, .07]	-.00 [-.06, .06]	-.42** [-.47, -.37]	-.33** [-.39, -.28]	-.28** [-.33, -.22]		
7. Between DMN-SLN	0.06	0.04	.02 [-.04, .09]	.01 [-.05, .07]	-.32** [-.38, -.27]	-.44** [-.49, -.39]	-.44** [-.49, -.39]	-.05 [-.11, .01]	
8. Between DMN-SMN	0.02	0.03	.03 [-.03, .10]	.02 [-.04, .08]	-.33** [-.39, -.28]	-.37** [-.42, -.31]	-.32** [-.37, -.26]	.11** [.05, .17]	.03 [-.03, .10]

FEMALES - *Means, standard deviations, and correlations with confidence intervals*

Variable	*M*	*SD*	1	2	3	4	5	6	7
1. BPM Total score	8.23	6.09							
2. SDSC Total score	36.59	7.40	.24** [.18, .30]						
3. Within SMN	0.28	0.03	-.03 [-.10, .03]	**-.08* [-.14, -.02]**					
4. Within DMN	0.26	0.03	.05 [-.01, .11]	-.01 [-.08, .05]	.05 [-.01, .11]				
5. Within SLN	0.29	0.03	-.01 [-.07, .06]	-.02 [-.08, .04]	-.08* [-.14, -.02]	.20** [.15, .26]			
6. Between SLN-SMN	0.09	0.03	.01 [-.05, .08]	.04 [-.02, .10]	-.31** [-.36, -.25]	-.34** [-.39, -.29]	-.28** [-.33, -.22]		
7. Between DMN-SLN	0.06	0.04	-.01 [-.07, .05]	.05 [-.01, .11]	-.41** [-.46, -.36]	-.41** [-.46, -.36]	-.42** [-.47, -.37]	-.12** [-.18, -.06]	
8. Between DMN-SMN	0.02	0.02	-.01 [-.07, .05]	.02 [-.04, .08]	-.27** [-.32, -.21]	-.36** [-.41, -.31]	-.31** [-.36, -.25]	.07* [.01, .13]	.05 [-.01, .11]

Correlations (with 95% confidence intervals in brackets) between network densities and symptom domains, significant at *$p < .05$, and **$p < .01$. Correlations between network densities and symptom domains are in black font, with significant associations in bold. Correlations between variables in the same domain are in gray. *M*: mean; *SD:*standard deviation.

© 2025 Hidalgo-Lopez E et al. *JAMA Network Open*.

eReferences.

1. Hansen BB. Full matching in an observational study of coaching for the SAT. *Journal of the American Statistical Association*. 2004;99(467):609-618.

2. Gu XS, Rosenbaum PR. Comparison of multivariate matching methods: Structures, distances, and algorithms. *Journal of Computational and Graphical Statistics*. 1993;2(4):405-420.

3. Scherrer KH, Ziadni MS, Kong J-T, et al. Development and validation of the Collaborative Health Outcomes Information Registry body map. *Pain reports*. 2021;6(1)

4. Ting TV, Barnett K, Lynch-Jordan A, Whitacre C, Henrickson M, Kashikar-Zuck S. 2010 American College of Rheumatology adult fibromyalgia criteria for use in an adolescent female population with juvenile fibromyalgia. *The Journal of pediatrics*. 2016;169:181-187. e1.

5. Achenbach TM, McConaughy S, Ivanova M, Rescorla L. Manual for the ASEBA brief problem monitor (BPM). *Burlington, VT: ASEBA*. 2011;33

6. Piper BJ, Gray HM, Raber J, Birkett MA. Reliability and validity of brief problem monitor, an abbreviated form of the child behavior checklist. *Psychiatry and clinical neurosciences*. 2014;68(10):759-767.

7. Bruni O, Ottaviano S, Guidetti V, et al. The Sleep Disturbance Scale for Children (SDSC) Construct ion and validation of an instrument to evaluate sleep disturbances in childhood and adolescence. *Journal of sleep research*. 1996;5(4):251-261.

8. Petersen AC, Crockett L, Richards M, Boxer A. A self-report measure of pubertal status: Reliability, validity, and initial norms. *J Youth Adolesc*. Apr 1988;17(2):117-33. doi:10.1007/BF01537962

9. Veale JF. Edinburgh Handedness Inventory - Short Form: a revised version based on confirmatory factor analysis. *Laterality*. 2014;19(2):164-77. doi:10.1080/1357650X.2013.783045

10. Kaplan CM, Schrepf A, Mawla I, et al. Neurobiological antecedents of multisite pain in children. *Pain*. Apr 1 2022;163(4):e596-e603. doi:10.1097/j.pain.0000000000002431

11. Ichesco E, Schmidt-Wilcke T, Bhavsar R, et al. Altered resting state connectivity of the insular cortex in individuals with fibromyalgia. *The journal of pain*. 2014 Aug 2014;15(8)doi:10.1016/j.jpain.2014.04.007

12. Suñol M, Payne MF, Tong H, et al. Reduced Resting-State Connectivity in Sensory Regions in Juvenile Fibromyalgia. *The Journal of Pain*. 2022;23(5):42-43.

13. Bhatt RR, Gupta A, Labus JS, et al. Altered Brain Structure and Functional Connectivity and Its Relation to Pain Perception in Girls With Irritable Bowel Syndrome. *Psychosom Med*. Feb/Mar 2019;81(2):146-154. doi:10.1097/PSY.0000000000000655

14. Sunol M, Payne MF, Tong H, et al. Brain Structural Changes During Juvenile Fibromyalgia: Relationships With Pain, Fatigue, and Functional Disability. *Arthritis Rheumatol*. Jul 2022;74(7):1284-1294. doi:10.1002/art.42073

15. Schmidt-Wilcke T, Ichesco E, Hampson JP, et al. Resting state connectivity correlates with drug and placebo response in fibromyalgia patients. *Neuroimage Clin*. 2014;6:252-61. doi:10.1016/j.nicl.2014.09.007

16. Seminowicz DA, Moayedi M. The Dorsolateral Prefrontal Cortex in Acute and Chronic Pain. *J Pain*. Sep 2017;18(9):1027-1035. doi:10.1016/j.jpain.2017.03.008

17. Tong H, Maloney TC, Payne MF, et al. Processing of pain by the developing brain: evidence of differences between adolescent and adult females. *Pain*. Sep 1 2022;163(9):1777-1789. doi:10.1097/j.pain.0000000000002571

© 2025 Hidalgo-Lopez E et al. *JAMA Network Open*.

18. Kutch JJ, Ichesco E, Hampson JP, et al. Brain signature and functional impact of centralized pain: a multidisciplinary approach to the study of chronic pelvic pain (MAPP) network study. *Pain*. Oct 2017;158(10):1979-1991. doi:10.1097/j.pain.0000000000001001

19. Segerdahl AR, Mezue M, Okell TW, Farrar JT, Tracey I. The dorsal posterior insula subserves a fundamental role in human pain. *Nature neuroscience*. 2015;18(4):499-500.

20. Baliki MN, Petre B, Torbey S, et al. Corticostriatal functional connectivity predicts transition to chronic back pain. *Nat Neurosci*. Jul 1 2012;15(8):1117-9. doi:10.1038/nn.3153

21. Hubbard CS, Becerra L, Heinz N, et al. Abdominal Pain, the Adolescent and Altered Brain Structure and Function. *PLoS One*. 2016-05-31 2016;11(5):e0156545. doi:10.1371/journal.pone.0156545

22. Saha R, Saha DK, Rahaman MA, Fu Z, Calhoun VD. Longitudinal Whole-Brain Functional Network Change Patterns Over A Two-Year Period In The ABCD Data. *IEEE*; 2022:1-4.

23. Thomason ME, Dennis EL, Joshi AA, et al. Resting-state fMRI can reliably map neural networks in children. *Neuroimage*. Mar 1 2011;55(1):165-75. doi:10.1016/j.neuroimage.2010.11.080

24. Marusak HA, Calhoun VD, Brown S, et al. Dynamic functional connectivity of neurocognitive networks in children. *Hum Brain Mapp.* Jan 2017;38(1):97-108. doi:10.1002/hbm.23346

25. Thomason ME, Chang CE, Glover GH, Gabrieli JD, Greicius MD, Gotlib IH. Default-mode function and task-induced deactivation have overlapping brain substrates in children. *Neuroimage*. Jul 15 2008;41(4):1493-503. doi:10.1016/j.neuroimage.2008.03.029

© 2025 Hidalgo-Lopez E et al. *JAMA Network Open*.
