# Supplementary material for: Sex, Neural Networks, and Behavioral Symptoms Among Adolescents With Multisite Pain
Source: JAMA Netw Open. 2025 Apr 16;8(4):e255364. doi: 10.1001/jamanetworkopen.2025.5364 (PMC12004202; doi:10.1001/jamanetworkopen.2025.5364)
Supplement: Supplement 2. — Data Sharing Statement [file jamanetwopen-e255364-s002.pdf]

# Data Sharing Statement

Hidalgo-Lopez. Sex, Neural Networks, and Behavioral Symptoms Among Adolescents With Multisite Pain. *JAMA Netw Open*. Published April 16, 2025.
doi:10.1001/jamanetworkopen.2025.5364

**Data**
**Data available:** Yes
**Data types:** Other (please specify)
**Additional Information:** The deidentified data and a data dictionary are already openly available with completion of a data use agreement through the ABCD Study release 4.0, NDA Study #1299, DOI: 10.15154/1523041.
**How to access data:** https://nda.nih.gov/study.html?id=1299
**When available:** beginning date: 08-21-2024

**Supporting Documents**
**Document types:** None

**Additional Information**
**Who can access the data:** The data are already openly available with completion of a data use agreement through the ABCD Study release 4.0, NDA Study #1299, DOI: 10.15154/1523041.
**Types of analyses:** The data are already openly available with completion of a data use agreement through the ABCD Study release 4.0, NDA Study #1299, DOI: 10.15154/1523041.
**Mechanisms of data availability:** The data are already openly available with completion of a data use agreement through the ABCD Study release 4.0, NDA Study #1299, DOI: 10.15154/1523041.
